# Exploration of Serum Proteomic Profiling and Diagnostic Model That Differentiate Crohn's Disease and Intestinal Tuberculosis

**DOI:** 10.1371/journal.pone.0167109

**Published:** 2016-12-20

**Authors:** Fenming Zhang, Chengfu Xu, Longgui Ning, Fengling Hu, Guodong Shan, Hongtan Chen, Ming Yang, Wenguo Chen, Jiekai Yu, Guoqiang Xu

**Affiliations:** 1 Department of Gastroenterology, The First Affiliated Hospital of Zhejiang University School of Medicine, Hangzhou, Zhejiang Province, China; 2 Department of Tumor Research Institute, The Second Affiliated Hospital of Zhejiang University School of Medicine, Hangzhou, Zhejiang Province, China; CHA University, REPUBLIC OF KOREA

## Abstract

**Aim:**

To explore the diagnostic models of Crohn’s disease (CD), Intestinal tuberculosis (ITB) and the differential diagnostic model between CD and ITB by analyzing serum proteome profiles.

**Methods:**

Serum proteome profiles from 30 CD patients, 21 ITB patients and 30 healthy controls (HCs) were analyzed by using weak cationic magnetic beads combined with MALDI-TOF-MS technique to detect the differentially expressed proteins of serum samples. Three groups were made and compared accordingly: group of CD patients and HCs, group of ITB patients and HCs, group of CD patients and ITB patients. Wilcoxon rank sum test was used to screen the ten most differentiated protein peaks (*P* < 0.05). Genetic algorithm combining with support vector machine (SVM) was utilized to establish the optimal diagnostic models for CD, ITB and the optimal differential diagnostic model between CD and ITB. The predictive effects of these models were evaluated by Leave one out (LOO) cross validation method.

**Results:**

There were 236 protein peaks differently expressed between group of CD patients and HCs, 305 protein peaks differently expressed between group of ITB patients and HCs, 332 protein peaks differently expressed between group of CD patients and ITB patients. Ten most differentially expressed peaks were screened out between three groups respectively (*P* < 0.05) to establish diagnostic models and differential diagnostic model. A diagnostic model comprising of four protein peaks (M/Z 4964, 3029, 2833, 2900) can well distinguish CD patients and HCs, with a specificity and sensitivity of 96.7% and 96.7% respectively. A diagnostic model comprising four protein peaks (M/Z 3030, 2105, 2545, 4210) can well distinguish ITB patients and HCs, with a specificity and sensitivity of 93.3% and 95.2% respectively. A differential diagnostic model comprising three potential biomarkers protein peaks (M/Z 4267, 4223, 1541) can well distinguish CD patients and ITB patients, with a specificity and sensitivity of 76.2% and 80.0% respectively. Among the eleven protein peaks from the diagnostic models and differential diagnostic model, two have been successfully purified and identified, Those two peaks were M/Z 2900 from the diagnostic model between CD and HCs and M/Z 1541 from the differential diagnostic model between CD and ITB. M/Z 2900 was identified as appetite peptide, M/Z 1541 was identified as Lysyl oxidase-like 2 (LOXL-2).

**Conclusion:**

The differently expressed protein peaks analyzed by serum proteome with weak cationic magnetic beads combined MALDI-TOF-MS technique can effectively distinguish CD patients and HCs, ITB patients and HCs, CD patients and ITB patients. The diagnostic model between CD patients and HCs consisting of four protein peaks (M/Z 4964, 3029, 2833, 2900), the diagnostic model between ITB patients and HCs comprising four protein peaks (M/Z 3030, 2105, 2545, 4210) and the differential diagnostic model between CD patients and ITB patients comprising three protein peaks (M/Z 4267, 4223, 1541) had high specificity and sensitivity and can contribute to diagnoses of CD, ITB and the differential diagnosis between CD and ITB. Two proteins from the diagnostic model of CD and the differential diagnostic model between CD and ITB were identified. Further experiments are required using a larger cohort of samples.

## Introduction

Crohn’s disease (CD) and Intestinal tuberculosis (ITB) are chronic inflammatory intestinal diseases^[^^1^^–^^4^^]^, it is difficult to differentiate them due to their high similarity in clinical manifestations, morphological features and histological characteristics^[^^1^^–^^4^^]^. Both CD and ITB have symptoms like abdominal pain, diarrhea, fever and weight loss, leading to low specificity of clinical manifestations. It is believed that appearence of longitudinal ulcers, aphthous ulcers and a cobblestone-like appearance are seen in typical CD with an endoscopy, and linear or circular ulcers are more inclined to the diagnosis of ITB^[^^5^^–^^7^^]^, however, in clinical practice, those findings are often atypical and with low positive rates. Both CD and ITB show granulomatous inflammation pathologically, though caseous granulomas is regarded as the gold standard for the diagnosis of ITB^[^^8^^]^, but due to low positive rate^[^^9^^,^^10^^]^, the differential diagnosis between CD and ITB becomes really difficult^[^^11^^,^^12^^]^. However, the treatment, prognosis and natural history of these two entities are quite different. So, it is necessary to discover new specific biomarkers which can differentiate CD from ITB.

Serum proteomic fingerprint, which aims to analyze the proteome signature of each patient, although technically difficult, could be a realistic approach to understand the biological processes involved in inflammatory processes^[^^13^^,^^14^^]^. It is a new type of proteomics technology developed in recent years, aiming to explore disease-related proteins, study protein expression or change after modification, look for diagnostic markers and establish diagnostic models^[^^15^^,^^16^^]^. Matrix-assisted laser desorption/ionization time-of-flight mass spectrometry (MALDI-TOF-MS) is an important proteomic technology. Many protein biomarkers of certain diseases have been indicated by using MALDI-TOF MS to analyze the serum proteome. Magnetic beads have large surface area and can capture more small molecular peptides and proteins^[^^17^^,^^18^^]^. The combination of MALDI-TOF-MS and magnetic beads can take advantages of both and therefore detect more low molecular weight proteins in serum. Few research used this technology to detect potentially useful markers that can help differentiating ITB and CD. In this study, we used weak cationic magnetic beads combined with MALDI-TOF-MS technique to analyze serum proteome of CD patients, ITB patients and healthy controls (HCs). Based on the genetic algorithm combined with support vector machine (SVM) model, we selected the best diagnostic models and differential diagnostic models which were validated with Leave one out (LOO) method, expecting to provide new ideas about the early diagnoses of CD, ITB and the differential diagnosis between CD and ITB at serum protein level.

## Materials and Methods

### Patients

Patients with CD and ITB at the Department of Gastroenterology, the First Affiliated Hospital of Zhejiang University School of Medicine were enrolled from December 2011 to December 2014. The diagnosis of CD was made by gastroenterologists specialized in Inflammatory Bowel Disease (IBD), based on the European Crohn’s and Colitis Organization guideline which is a combined evaluation of clinical, endoscopic and histological features^[^^19^^]^. The diagnostic criteria of ITB is a combination of clinical manifestations, endoscopic presentation, histological features, micro-biological tests results and response to anti-tuberculous treatment^[^^20^^,^^21^^]^. This study was approved by the Ethics committee of the First Affiliated Hospital of Zhejiang University School of Medicine (Reference Number: 2014–303) and informed consent was obtained from all patients. It was an observational study and verbal informed consent was received from every participant. All patients were in inpatients department and undergone routine blood examination during hospital stay, so when the nursing staff performed blood drawing for every patient for the first time, there was a specialist consulting with patients’ willingness of using their specimens in this study, we collected venous blood from those who were willing to use their serum specimens in our research. Subjects without any familial disposition for IBD, daily medication, or any known diseases and did healthy check-up in this hospital at the corresponding period were selected as HCs. This study did no harm to the participants and the procedures were approved by the Ethics Committee of Zhejiang University School of Medicine. As shown in Table 1, totally, 30 CD patients (male/female 16/14, mean age 36.3±13.2, aged 21–61) and 21 ITB patients (male/female 11/10, mean age 42.1±14.6, aged 25–70) were enrolled. All the patients were newly diagnosed cases, CD patients had not received infliximab therapy or Acetazolamide (AZA)/ 6-Mercaptopurine (6-MP)/ Methotrexate (MTX) and ITB patients had not received anti-TB treatment. HCs were composed of 30 persons (male/female 15/15, mean age 38.5±13.9, aged 24–66). There was no significant difference in either age (*P* = 0.218) or gender ratio (*P* = 0.102) between three groups.

**Table 1:** Gender and age of the participants

Group	Male/female	Average age
**CD**	16/14	36.3±13.2 (21–61)
**ITB**	11/10	42.1±14.6 (25–70)
**HCs**	15/15	38.5±13.9 (24–66)

CD: Crohn’s disease. ITB: Intestinal tuberculosis. HCs: Healthy Controls.

### Collection and preservation of serum samples

Blood sample of about 2mL venous blood were collected in vacuum tubes without anticoagulant and centrifuged for 6 min, with a rotate speed of 3600rpm. Then, the serum on the upper layer was suck up and put into the eppendorf tubes and was preserved at −80°C for further analysis. Blood sample from only those patients were included when the diagnosis of CD or ITB was confirmed and the follow up was complete. There was no hemolysis of blood specimens, no upper layer grease and lower layer cells were suck up. The above-mentioned steps were finished within 2 hours and all the samples were freeze and thawed no more than twice.

### Separation of serum protein

#### Preparation of serum sample

5μL serum sample was took from the −80°C freezer and put into the sample tube which containing magnetic beads and magnetic beads in combination with buffer solution, after suction and blending, the sample tube was preserved indoor temperature.

#### Preparation of weak cationic magnetic beads

Firstly, 10μL magnetic beads in combination with buffer solution was put into the 200μL sample tube, then, 10μL magnetic beads and 5μL serum sample were added blended for suction and blending. Secondly, a magnetic separator was put into the sample tube and the suspended liquid was sucked after separation, then, 100μL magnetic bead cleaning buffer was put into the sample tube and the suspended liquid was sucked after separation. Repeat the above steps twice to ensure that the suspended liquid was completely sucked. Lastly, 5μL magnetic bead elution buffer and the magnetic separator were added, after complete separation of the magnetic beads and suspended liquid, the suspended fluid sample was moved into a new 10μL tube, and 5μL magnetic beads stable buffer was added for suction and blending.

#### Standard substance sample application

The standard substance was dissolved at the room temperature and mixed with 1μL standard substance and 10μL matrix. Then, 1μL mixed liquor was put at the standard position of Anchorchip target and dried at room temperature. The Anchorchip target was put into the mass spectrometer (microflex MALDI-TOF-MS, German), using LP-Clinprot method. Then, the protein peaks with a relative molecular mass was collected at a range of Dalton 1000–13000. The same sample was with the same crystallization point, 5–10 points at every crystallization point were collected and the protein map was generated. The average molecular weight deviation was less than 0.0001.

#### Serum sample application

1μL serum sample disposed with the magnetic bead was put at the standard position of Anchorchip target and dried at room temperature. Anchorchip target was put into the mass spectrometer, choosing linear model, the protein peak with a relative molecular mass at a range of Dalton 800–10000 was collected. The same sample was with the same crystallization point, 8 points were collected, accumulating to 400. The average molecular weight deviation is less than 0.0001.

### Data processing

Firstly, we uploaded the original mass spectrogram to the server and removed noise caused by mass spectrometer detector using wavelet transform. After removing the noise, we corrected the baseline of mass spectrometry. Then, we corrected the molecule weight value of the whole spectrogram, found the protein peaks with local extremum method and filtered protein peaks with signal-to-noise ratio, thus, the ratio of each protein peak appeared in every sample. Lastly, we homogenized the intensity.

### Mass spectra analysis and establishment of diagnostic models

Preliminary statistical analysis was performed using Biomarker Wizard 3.1 software (calculation of $\bar{X}$, S and *P* value), the corresponding protein peak intensity of each M/Z was showed as $\bar{X}$±S. Differentially expressed (*P* < 0.05) protein peaks were screened out with Wilcoxon rank-sum test. Genetic algorithm combining with SVM was utilized to establish the optimal diagnostic models for CD, ITB and the optimal differential diagnostic model between CD and ITB. Due to the limited number of samples, the separation of the samples into independent and training test sets was not possible, so the evaluation of the classification was limited to LOO cross validation to calculate sensitivity, specificity and accuracy. One number of spectra was removed from the training set, a classifier was generated using the remaining instances, and the performance of this classifier was evaluated by applying it to the left out instances and comparing classifier and true labels. The protein peak combination which showed the highest cut-off value predicted by SVM was regarded as the final candidate biomarkers. *P* < 0.05 was considered as statistically significant.

### Identification of the biomakers

The sequences of differential peptides were identified by using ultrafleXtreme MALDI-TOF/TOF directly. After calibrated by peptide standard (Bruker Daltonic, Germany), we used the reflection mode to detect the isotopic peaks, and turned to the lift model to detect the MS/MS fragment. The BioTools and mascot software was used to search the database.

## Results

### Serum protein fingerprint

**Fig 1:**
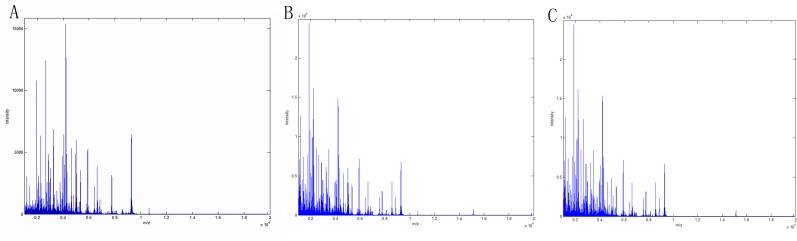
Overlay protein map of 3 groups. A Crohn’s disease patients vs Healthy controls. B Intestinal tuberculosis patients vs Healthy controls. C Crohn’s disease patients vs Intestinal tuberculosis patients

Serum protein peaks combined to weak cationic magnetic beads were detected by MALDI-TOF-MS. The serum protein peaks of 30 CD patients, 21 ITB patients and 30 HCs were shown in Fig 1.

### Screening of differentially expressed protein peaks

There were 236 differentially expressed (*P* < 0.05) serum protein peaks between 30 CD patients and 30 HCs, 305 differentially expressed serum protein peaks between 21 ITB and 30 HCs, 332 differentially expressed serum protein peaks between 30 CD patients and 21 ITB patients found by mass spectrometer. Ten most differentially expressed protein peaks were further chosed to establish diagnostic and differential diagnostic models, as shown in Tables 2–4. Among the ten most differentially expressed protein peaks between 30 CD patients and 30 HCs, six protein peaks (M/Z 4976, 4964, 4988, 2833, 2900, 4069) were highly expressed, four protein peaks (M/Z 3029, 3315, 6630, 5065) were lowly expressed (Table 2). Among the ten most differentially expressed protein peaks between 21 ITB patients and 30 HCs, one protein peak (M/Z 2545) was highly expressed, nine protein peaks (M/Z 3030, 2105, 5065, 1945, 1680, 3275, 4210, 6379, 4248) were lowly expressed (Table 3). Among the ten most differentially expressed protein peaks between 30 CD patients and 21 ITB patients, nine protein peaks (M/Z 4210, 4195, 4267, 2105, 2933, 4223, 6380, 2952, 4248) were highly expressed, one protein peak (M/Z 1541) was lowly expressed (Table 4).

**Table 2:** Ten differentially expressed serum protein peaks of CD patients and HCs ($\bar{X}$±S)

M/Z	$\bar{X}$±S		*P*
	CD(N = 30)	HCs(N = 30)	
4976	369.99±252.56	91.56±49.67	1.31*10^−8^
4964	1585.34±1255.30	446.25±225.48	7.70*10^−8^
3029	321.63±228.67	747.92±327.02	7.04*10^−7^
4988	108.34±51.78	42.37±28.54	8.20*10^−7^
3315	325.63±180.45	666.65±275.90	3.83*10^−6^
2833	135.66±81.02	50.74±43.03	1.02*10^−5^
6630	1019.33±436.15	1746.38±721.76	1.53*10^−5^
4069	299.74±151.92	160.36±71.69	2.77*10^−5^
2900	399.23±183.07	207.46±114.64	3.59*10^−5^
5065	232.41±251.78	505.92±262.69	3.59*10^−5^

CD: Crohn’s disease. HCs: Healthy Controls.

**Table 3:** Ten differentially expressed serum protein peaks of ITB patients and HCs ($\bar{X}$±S)

M/Z	$\bar{X}$±S		*P*
	ITB(N = 21)	HCs(N = 30)	
3030	254.30±214.62	828.02±352.65	2.03*10–7
2105	439.37±425.78	1325.04±443.44	3.75*10–7
2545	355.37±258.72	100.56±81.06	1.01*10–6
5065	117.29±182.40	544.69±303.70	1.97*10–6
1945	1128.58±1453.16	3689.98±1842.34	4.17*10–6
1680	56.62±41.68	162.02±81.39	1.03*10–5
3275	261.96±252.91	999.05±774.40	1.03*10–5
4210	3333.29±3034.13	7918.54±2663.17	1.03*10–5
6379	30.20±41.31	112.59±64.51	1.22*10–5
4248	185.86±170.31	419.39±177.54	2.24*10–5

ITB: Intestinal tuberculosis. HCs: Healthy Controls.

**Table 4:** Ten differentially expressed serum protein peaks of CD patients and ITB patients ($\bar{X}$±S)

M/Z	$\bar{X}$±S		*P*
	CD(N = 30)	ITB(N = 21)	
4210	8342.18±2548.39	3291.11±3046.65	3.46*10^−6^
4195	558.47±159.18	239.90±265.59	5.00*10^−6^
4267	853.94±489.67	335.87±234.22	6.57*10^−6^
2105	1185.54±417.36	433.09±428.49	7.86*10^−6^
2933	734.23±572.21	237.39±533.94	7.86*10^−6^
4223	787.90±329.38	326.99±266.34	1.22*10^−5^
6380	108.64±72.41	30.12±41.40	1.73*10^−5^
2952	681.89±416.57	276.08±425.83	6.59*10^−5^
4248	382.41±173.16	178.09±172.40	7.15*10^−5^
1541	70.37±56.20	153.44±70.42	9.83*10^−5^

CD: Crohn’s disease. ITB: Intestinal tuberculosis.

### Establishment of diagnostic models of CD, ITB and differential diagnostic model between CD and ITB

**Fig 2:**
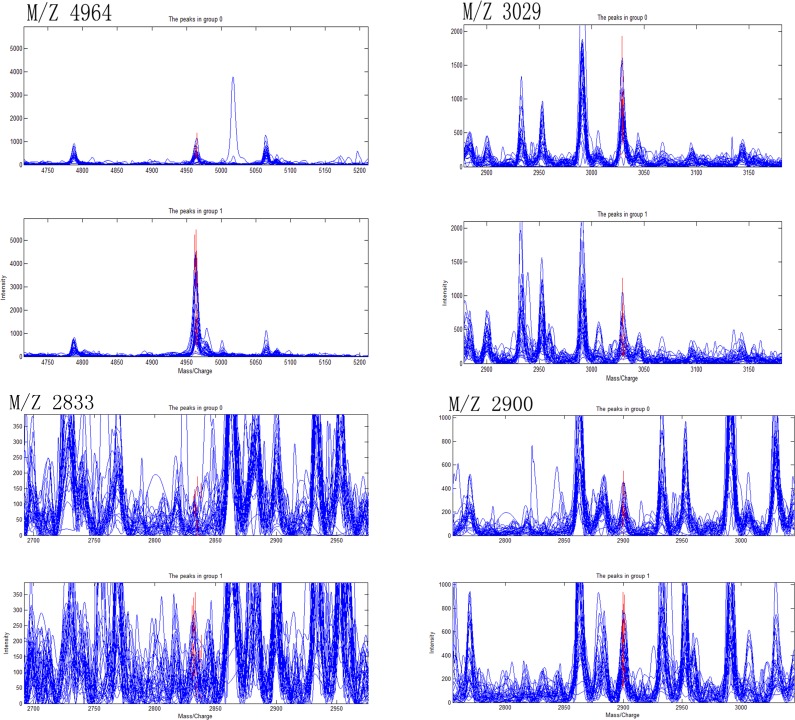
Four selected peaks (M/Z 4964, 3029, 2833, 2900) that compose of the SVM model for the diagnose of Crohn’s disease. Group 1 representatives Crohn’s disease patients, group 0 representatives Healthy controls.

**Fig 3:**
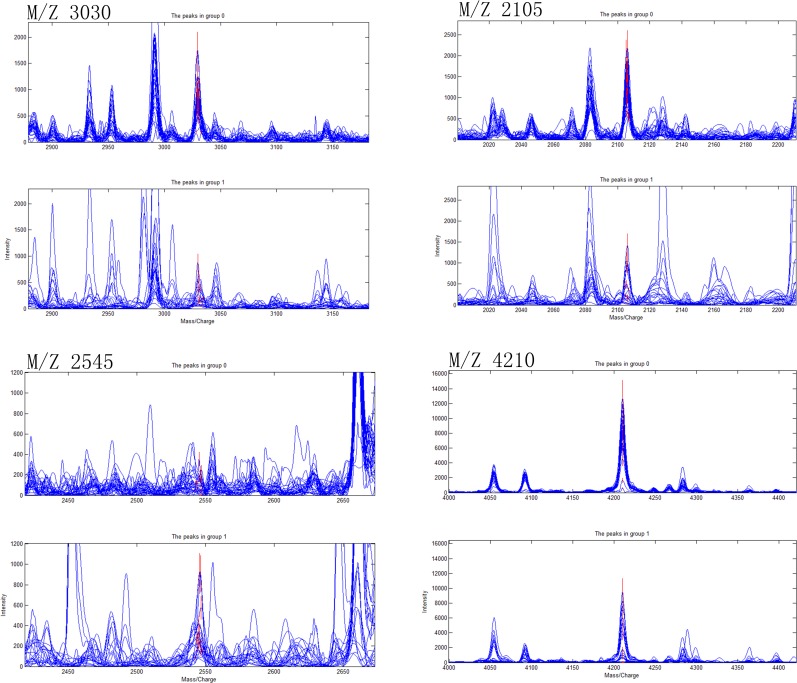
Four selected peaks (M/Z 3030, 2105, 2545, 4210) that compose of the SVM model for the diagnose of Intestinal tuberculosis. Group 1 representatives Intestinal tuberculosis patients, group 0 representatives Healthy controls.

**Fig 4:**
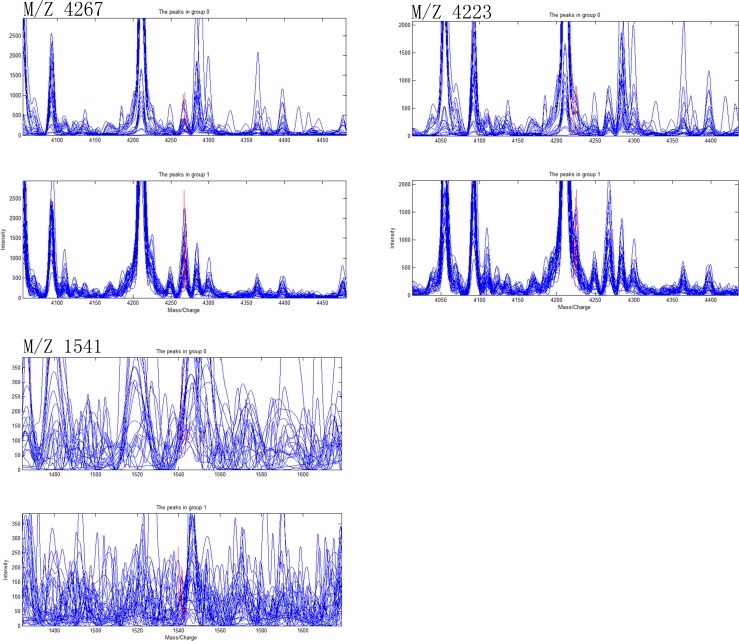
Three selected peaks (M/Z 4267, 4223, 1541) that compose of the SVM model for the differential diagnose between Crohn’s disease and Intestinal tuberculosis. Group 1 representatives Crohn’s disease patients, group 0 representatives Intestinal tuberculosis patients.

Ten most differentially expressed protein peaks of the three groups were chosed respectively to establish the optimal diagnostic models for CD, ITB and the optimal differential diagnostic model to distinguish CD from ITB using genetic algorithm combining with SVM. The predictive effects of these models were evaluated by LOO cross validation method. The protein peaks combination which showed the highest cut-off value predicted by SVM was regarded as the final candidate biomarkers combination (Figs 2–4).

**Fig 5:**
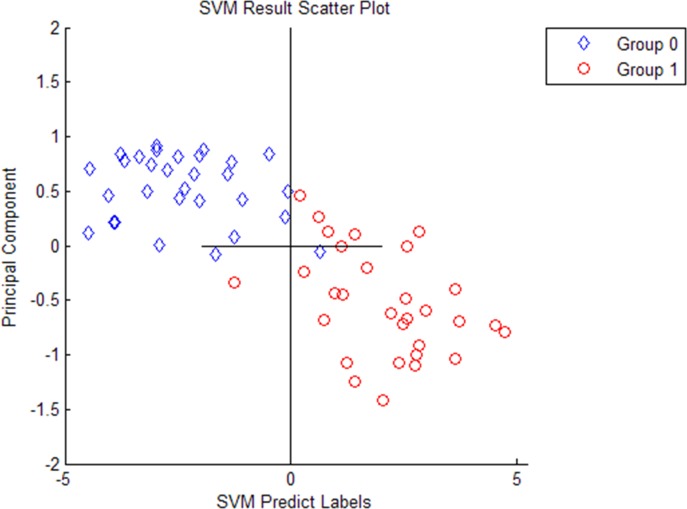
The discriminant results by SVM of Crohn’s disease patients vs Healthy controls. Group 1 represents Crohn’s disease patients, group 0 represents Healthy controls. Every hollow point indicates one sample.

**Fig 6:**
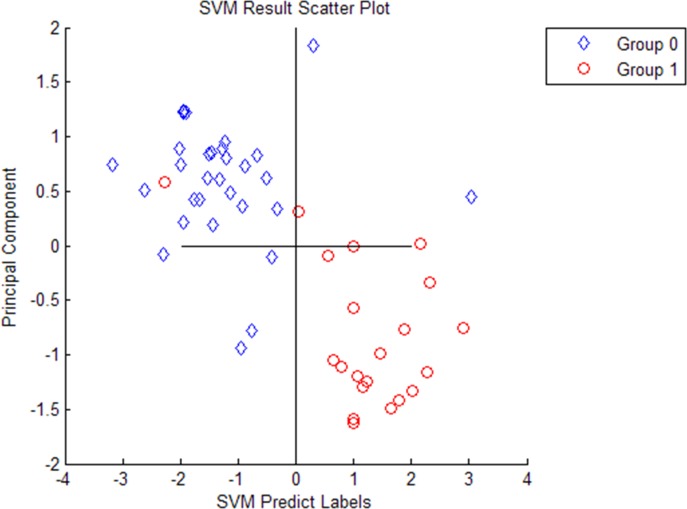
The discriminant results by SVM of Intestinal tuberculosis patients vs Healthy controls. Group 1 represents Intestinal tuberculosis patients, group 0 represents Healthy controls. Every hollow point indicates one sample.

**Fig 7:**
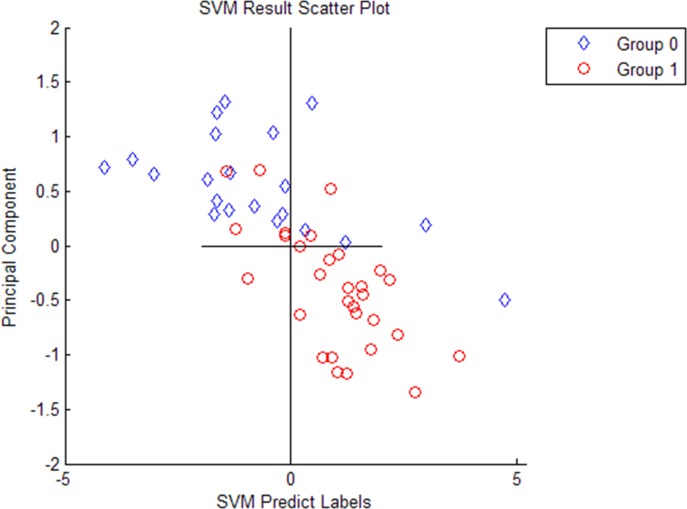
The discriminant results by SVM of Crohn’s disease patients vs Intestinal tuberculosis patients. Group 1 represents Crohn’s disease patients, group 0 represents Intestinal tuberculosis patients. Every hollow point indicates one sample.

The discriminant results by SVM of the three groups were obviously showed on the scatter diagram (Figs 5–7). We found a diagnostic model consisting of four protein peaks (M/Z 4964, 3029, 2833, 2900) can well distinguish CD patients and HCs. Among those four protein peaks, three protein peaks (M/Z 4964, 2833, 2900) were highly expressed and one protein peak (M/Z 3029) was lowly expressed in CD patients compared with that in HCs (Table 2). When using this diagnostic model to detect 30 CD patients and 30 HCs, 1 CD patient was misjudged as HC, and 1 HC was misjudged as CD patient, with a specificity and sensitivity of 96.7% and 96.7% respectively (Fig 5), indicating this diagnostic model had a high accuracy. A diagnostic model consisting of four protein peaks (M/Z 3030, 2105, 2545, 4210) can well distinguish ITB patients and HCs. Among those four protein peaks, one protein peak (M/Z 2545) was highly expressed and three protein peaks (M/Z 3030, 2105, 4210) were lowly expressed in ITB patients compared with that in HCs (Table 3). When using this diagnostic model to detect 21 ITB patients and 30 HCs, 1 ITB patient was misjudged as HC and 2 HCs were misjudged as ITB patients, with a specificity and sensitivity of 93.3% and 95.2% respectively (Fig 6), indicating this diagnostic model had a high accuracy. A differential diagnostic model consisting of three protein peaks (M/Z 4267, 4223, 1541) can well distinguish CD patients from ITB patients. Among the three protein peaks, two protein peaks (M/Z 4267, 4223) were highly expressed and one protein peak (M/Z 1541) was lowly expressed in CD patients compared with that in ITB patients (Table 4). When using this diagnostic model to detect 30 CD patients and 21 ITB patients, 6 CD patients were misjudged as ITB patients, and 5 ITB patients were misjudged as CD patients, with a specificity and sensitivity of 76.2% and 80% respectively (Fig 7), indicating this differential diagnostic model had a relatively high accuracy. The specificity, sensitivity, positive predictive value and negative predictive value of the diagnostic models of CD, ITB and the differential diagnostic between CD and ITB were shown in Table 5.

**Table 5:** Validation results of the diagnostic models

Groups	N	Sensitivity(%)	Specificity(%)	Positive predictive value(%)	Negative predictive value(%)
**CD/HCs**	30/30	96.7	96.7	96.7	96.7
**ITB/HCs**	21/30	95.2	93.3	90.9	96.6
**CD/ITB**	30/21	80.0	76.2	82.3	72.7

CD: Crohn’s disease. ITB: Intestinal tuberculosis. HCs: Healthy Controls.

### Identification of the biomarkers

Among the eleven protein peaks of the diagnostic models and differential diagnostic model, two have been successfully purified and identified. They two peaks were M/Z 2900 from the diagnostic model between CD and HCs and M/Z 1541 from the differential diagnostic model between CD and ITB. Table 6 showed the Mascot Search Results of the three protein peaks. M/Z 2900 was identified as appetite peptide, M/Z 1541 was identified as LOXL-2.

**Table 6:** Mascot Search Results of the identified protein peaks

SwissProt Accession No	*P*	Protein Name	Peptide
GHRL_HUMAN	3.7*10^−2^	Appetite peptide	N.SPSSTGSGNTEHSCSS.Q
LOXL2_HUMAN	3.6*10^−2^	LOXL-2	T.LAACTSNGWGVTDCK.H

Lysyl oxidase-like 2: LOXL-2

## Discussion

Proteomic analysis, as a new comprehensive technology used for the identification of biomarkers, has been widely used in many fields since 1994. There are more than 20000 kinds of proteins in serum, which can reflect the serological changes of different stages for different diseases^[^^22^^–^^25^^]^. Using proteomics technology to screening specific proteins can provide new ideas and methods for the early diagnosis, treatment and prognosis of diseases. MALDI-TOF-MS technology is a newly developing kind of proteomics research methods in recent years, with a theory that the laser radiates the crystals comprised of sample and matrix, leading to sample ionization, ionized sample fly accelerated through pipes with the effect of electric field, by detecting the flight time to the detector, M/Z is measured. This method, with advantages of low sample consumption, easy operation, high resolution, good repeatability and high sensitivity, can detect and differentiate polypeptides containing diagnostic message and obtain polypeptide biological information with high sensitivity^[^^26^^–^^28^^]^. When detecting low abundance proteins with small relative molecular mass, the effects are more apparent. Compared with surface-Enhanced Laser Desorption/ Ionization-Time of Fight-Mass Spectrometry (SELDI-TOF-MS) technology, the difference of MALDI-TOF-MS lies in that it replace chip with weak cationic magnetic beads which has a better binding force to separate protein^[^^29^^]^.

Borrowed from the calculation model of the natural selection of Darwin's biological theory and the biological evolution process of genetic mechanism, genetic algorithm simulates the natural evolution process and searches the optimal solution method through mechanism of natural selection, heredity and mutation, which is very effective for combinatorial optimization problems^[^^30^^,^^31^^]^. SVM method, based on limited sample information, aims to seek the best compromise between the complexity of the model (accuracy of the specific training samples) and the learning ability (the ability to identify any sample without error) to get the best generalization ability.

This study adopted the strategy of combination of filtering method which uses Wilcoxon rank sum test and model dependent method, filtered out protein peaks without significant difference and made model dependent screening for protein peaks with significant difference. The benefits of this strategy are that it overcomes the defect of a relatively low model accuracy which established by filtering method and overcome shortcomings like large amount of calculation and time-consuming which exists in model dependent method. Another problem is how to verify the accuracy of the models involving sample classification, that is, how many samples are divided into training set to build the model, how many samples are divided into test set to verify the model and evaluate the accuracy of the model. There are three methods to validate model accuracy: dichotomy, 10 times crossover method and LOO method. Here, we used LOO method.

The differential diagnosis between CD and ITB is difficult. Firstly, they have a lot in common in endoscopic and pathological manifestations, with granulomatous inflammation as the common pathological features. Features like submucosal inflammation, aphthous ulcer, annular ulcer, expansion of ileocecal junction, granuloma and granulation tissue hyperplasia are hard to differentiate these two diseases. Therefore, the pathological diagnoses for both of them are huge challenges even for experienced pathologists. Secondly, diagnostic criterial for ITB contain acid fast stain and caseous necrosis are often difficult to be found, with a positive rate of only 18.33% and the positive rate of cultivation for mycobacterium tuberculosis is less than 20%^[^^32^^–^^34^^]^. However, the treatment for CD and ITB are widely divergent, especially glucocorticoid which is an important treatment for CD may cause focal diffusion, if was used mistakenly for ITB. The prognosis for this two diseases are totally different, CD, instead of being cured, is usually controlled in a stable state and easy to recur. But for ITB, once with early confirmed diagnosis and timely treatment, it can be completely cured. Although there are some studies at home and abroad have found that regression mathematical model of clinical indicators^[^^35^^]^, anus rectum disease^[^^36^^]^, tuberculosis, anal fistula, parenteral performance^[^^37^^]^ can help differentiating CD and ITB, however, no specific serological biomarkers can differentiate these two diseases yet. Lokesh A, et al performed proteomics analysis of mucosal biopsies from patients with five CD and five ITB patients, they chose six differentially expressed proteins for validation using immunohistochemistry in a larger cohort of patients, but none of them was differentially expressed in patients with ITB and CD, and they did not establish a diagnostic models for CD, ITB or the differential diagnostic model between CD and ITB.

The present study is the first ever proteomics analysis of serum biopsies from patients with CD and ITB and it has revealed several promising biomarker candidates for differentiation between these two diseases. We firstly analyzed the serum protein profiling of 30 CD patients, 21 ITB patients and 30 HCs by using MALDI-TOF-MS technology, and found that there were 236 differently expressed protein peaks between CD patients and HCs, 305 differently expressed protein peaks between ITB patients and HCs, 332 differently expressed protein peaks between CD patients and ITB patients, indicating that the serum protein profiling between CD and ITB, CD and HCs, ITB and HCs have a lot of differences. The mass spectra analysis revealed a large number of potentially interesting protein peaks with significant *P* value. We chose ten most differentially expressed protein peaks of each group, and used genetic algorithm combining with SVM to establish a diagnostic model of CD (combination of four protein peaks of M/Z 4964, 3029, 2833, 2900, with a specificity and sensitivity of 96.7% and 96.7% respectively), a diagnostic model of ITB (combination of four protein peaks of M/Z 3030, 2105, 2545, 4210, with a specificity and sensitivity of 93.3% and 95.2% respectively), a differential diagnostic model distinguishing CD from ITB (combination of three protein peaks of M/Z 4267, 4223, 1541, with a specificity and sensitivity of 76.2% and 80.0% respectively), demonstrating that the diagnostic models consisting of several markers had great value in the diagnosis of CD, ITB and differential diagnosis between them. As these two entities are complex and heterogeneous, combination of several biomarkers may be more relevant for their diagnosis and differential diagnosis between them.

We achieved purification and identification of two relevant biomarkers (*P* < 0.05): Appetite peptide and LOXL-2. They are all known proteins and were reported in many studies. Dixit V.D, et al found that appetite peptide could control immune cell activation and inflammation by inhibiting the expression of proinflammatory anorectic cytokines such as IL-1β, IL-6, and TNF-α^[^^38^^]^. Studies also showed that appetite peptide could inhibit FGF-2-mediated angiogenesis in vitro and in vivo and was involved in protecting cardiomyocytes from apoptosis^[^^39^^].^ Mazzocchi G elaborated that appetite peptide enhanced the growth of cultured human adrenal zona glomerulosa cells by exerting MAPK-mediated proliferogenic and antiapoptotic effects^[^^40^^]^. A recent research showed that enhanced enteroendocrine cells responses may directly and adversely affect appetite in CD patients through increased gut-brain signaling by reducing appetite peptide^[^^41^^]^, but another study discovered that CD itself has no significant influence on circulating appetite peptide levels in the outpatients^[^^42^^]^. However, we found that appetite peptide distribution was higher in CD patients when compared with healthy controls, but due to the limited number of samples, we did not validated appetite peptide in a larger sample. Next, this step should be performed and the definite mechanism behind need to be explored. LOXL-2 was suggested to be specifically involved in cell adhesion and senescence in earlier study and recently, it was proved as a role in regulating angiogenesis through collagen IV scaffolding^[^^43^^]^ and to participated in the mechanisms in many tumors and carcinoma, like the effect on proliferation of hepatocellular carcinoma, mediating transition and proliferative growth of dormant tumor cells, and drive lung cancer invasion and metastasis^[^^44^^–^^46^^]^. However, there was no report of the role of LOXL2 in the pathogenesis of CD or ITB, and we found that LOXL2 distribution was lower in CD patients when compared with ITB, but due to the limited number of samples, we did not validated LOXL2 in a larger sample and the mechanisms warrant further investigation in the future.

This study is an exploratory research to achieve preliminary proteomic experiments on a panel of CD and ITB cases and HCs in order to obtain general diagnostic tools and differential diagnostic tool as well as general information concerning pathophysiology of these diseases. It has some limitations. Firstly, the sample size was relatively small and we selected both clinically active and inactive CD patients, ignoring the fact that important discriminating biomarkers, among which acute phase reactant proteins, might appear significant only in clinically active diseases. It also limits the evaluation of classification to LOO cross validation performance without the independent validation of the test set. In the next step, studies of larger numbers of patients are needed to validate these results on the independent sample cohorts, to analyze between subgroups of active and inactive CD patients and compare from two aspects: serum specimens and tissue samples for proteomics analysis to improve the credibility and specificity of the results. Secondly, validation of those diagnostic models and differential diagnostic model is necessary in larger sample size to confirm the value of these models in the diagnosis of CD, ITB and the differential diagnosis between CD and ITB. Thirdly, purification and identification of relevant biomarkers to make clear the corresponding peptides and proteins is indispensable to understand the etiology and pathogenesis of those diseases. Besides, although protein fingerprint has the advantages such as fast and high throughput, easy to standardization, it do has uncertainty of sample preparation method, protein separation, data acquisition and analysis method, which influences the results of mass spectrum fingerprint classification^[^^47^^]^. So efforts from clinical experts, personnel of mass spectrum technology research and development projects and bioinformatics experts are necessary to make MALDI-TOF-MS technique get widely used and long-term development.

To sum up, this study, using MALDI-TOF-MF technology, screened out differentially expressed protein peaks from serum samples between CD patients and HCs, ITB patients and HCs, CD patients and ITB patients. The diagnostic model of CD, IBD and the differential diagnostic model between CD and ITB established by genetic algorithm and SVM model has high specificity and sensitivity. Researching those protein peaks in-depth will contribute to further understanding of the etiology and pathogenesis of CD, will provide new methods for the diagnoses of CD and the differential diagnosis between CD and ITB.

## Conclusion

The differently expressed protein peaks analyzed by serum proteome with weak cationic magnetic beads combined MALDI-TOF-MS technique can effectively distinguish CD patients, ITB patients and healthy controls. By genetic algorithm combining with SVM, the diagnostic model between CD and HCs consisting of four protein peaks (M/Z 4964, 3029, 2833, 2900), the diagnostic model between ITB and HCs comprising four protein peaks (M/Z 3030, 2105, 2545, 4210) and the differential diagnostic model between CD and ITB comprising three potential biomarkers (M/Z 4267, 4223, 1541) had high specificity and sensitivity to the diagnoses of CD, ITB and the differential diagnosis between CD and ITB.

## Supporting Information

S1 File Serum proteomic profiling between CD and HCs. The biomarkers principal component scatter plot, the gel figure and peak figure of the ten most differentially expressed peaks and the results by using genetic algorithm combining with SVM.
(PDF)

S2 File Serum proteomic profiling between ITB and HCs. The biomarkers principal component scatter plot, the gel figure and peak figure of the ten most differentially expressed peaks and the results by using genetic algorithm combining with SVM.
(PDF)

S3 File Serum proteomic profiling between CD and ITB. The biomarkers principal component scatter plot, the gel figure and peak figure of the ten most differentially expressed peaks and the results by using genetic algorithm combining with SVM.
(PDF)
