# Correction: Exploration of Serum Proteomic Profiling and Diagnostic Model That Differentiate Crohn’s Disease and Intestinal Tuberculosis

**DOI:** 10.1371/journal.pone.0212300

**Published:** 2019-02-07

**Authors:** Fenming Zhang, Chengfu Xu, Longgui Ning, Fengling Hu, Guodong Shan, Hongtan Chen, Ming Yang, Wenguo Chen, Jiekai Yu, Guoqiang Xu

In the Funding section, the name of the funder is listed incorrectly. The correct funder is: Medical Science Research Foundation of Health Bureau of Zhejiang Province.
