# Supplementary material for: Exploration of Serum Proteomic Profiling and Diagnostic Model That Differentiate Crohn's Disease and Intestinal Tuberculosis
Source: PLoS One. 2016 Dec 20;11(12):e0167109. doi: 10.1371/journal.pone.0167109 (PMC5173341; doi:10.1371/journal.pone.0167109)
Supplement: S1 File — The biomarkers principal component scatter plot, the gel figure and peak figure of the ten most differentially expressed peaks and the results by using genetic algorithm combining with SVM. (PDF) [file pone.0167109.s001.pdf]

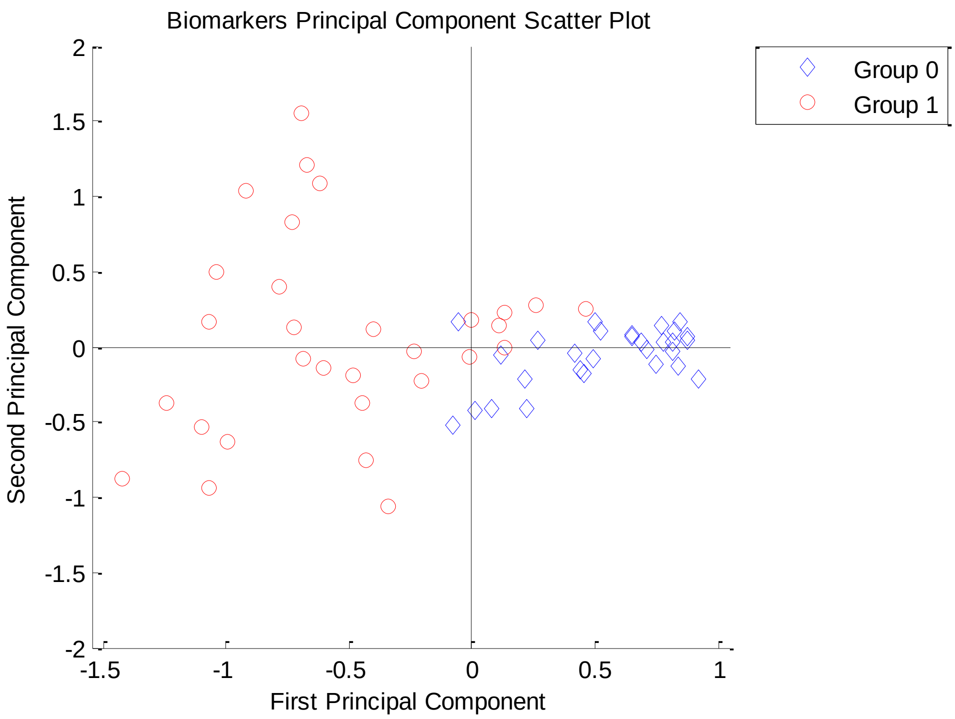

Biomarkers Principal Component Scatter Plot
Group 1: Crohn's disease patients, group 0: Healthy controls.

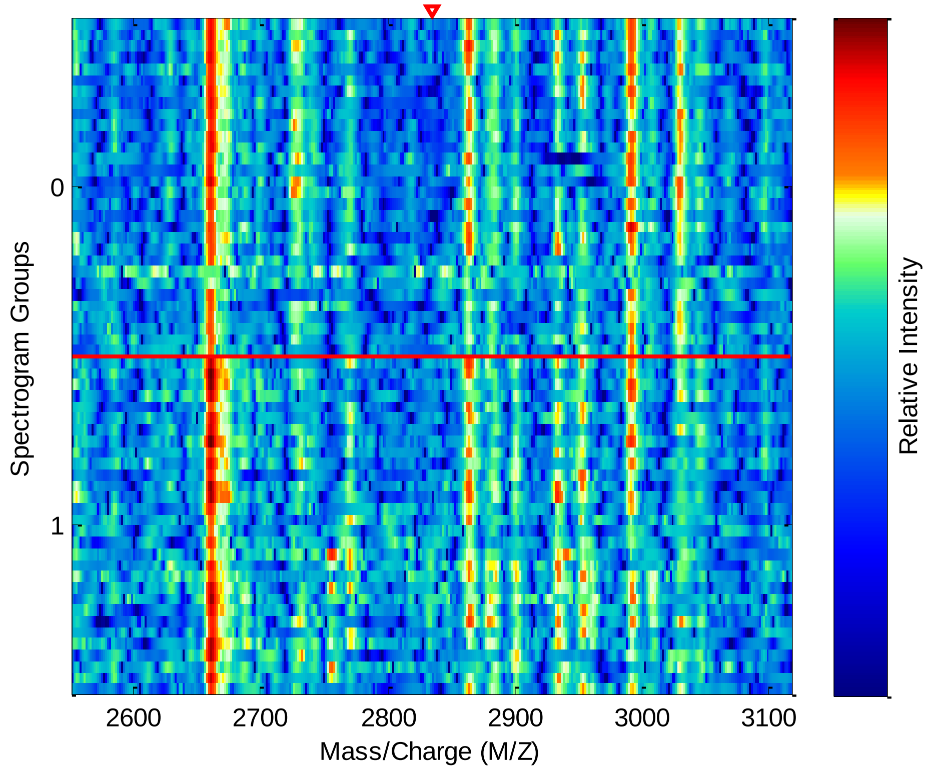

Gel figure: M/Z 2833

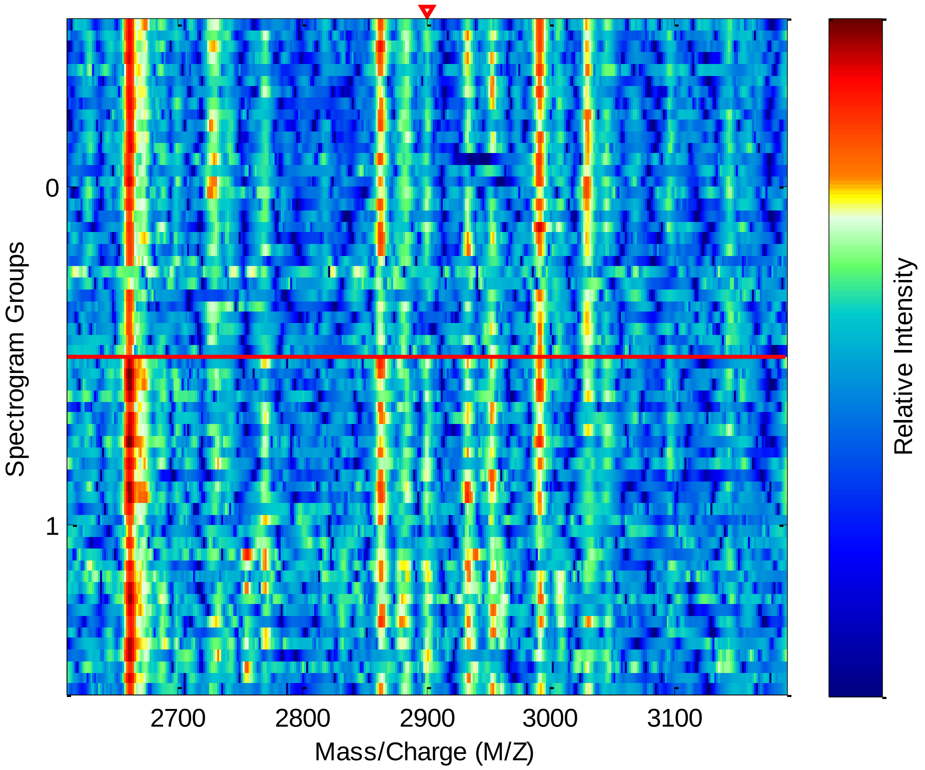

Gel figure: M/Z 2900

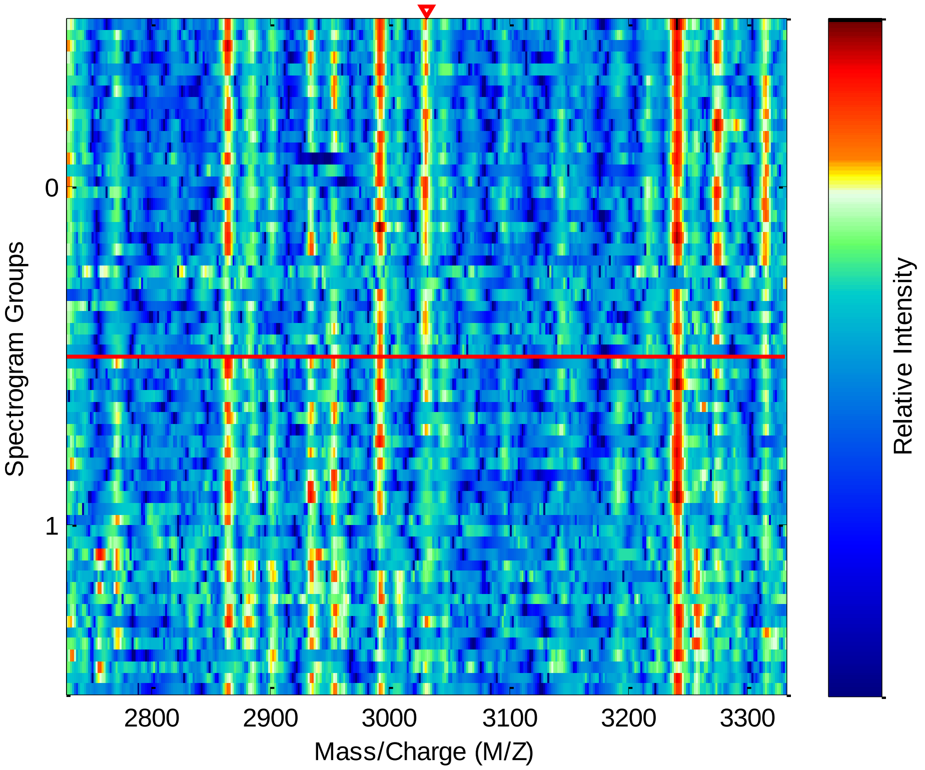

Gel figure: M/Z 3029

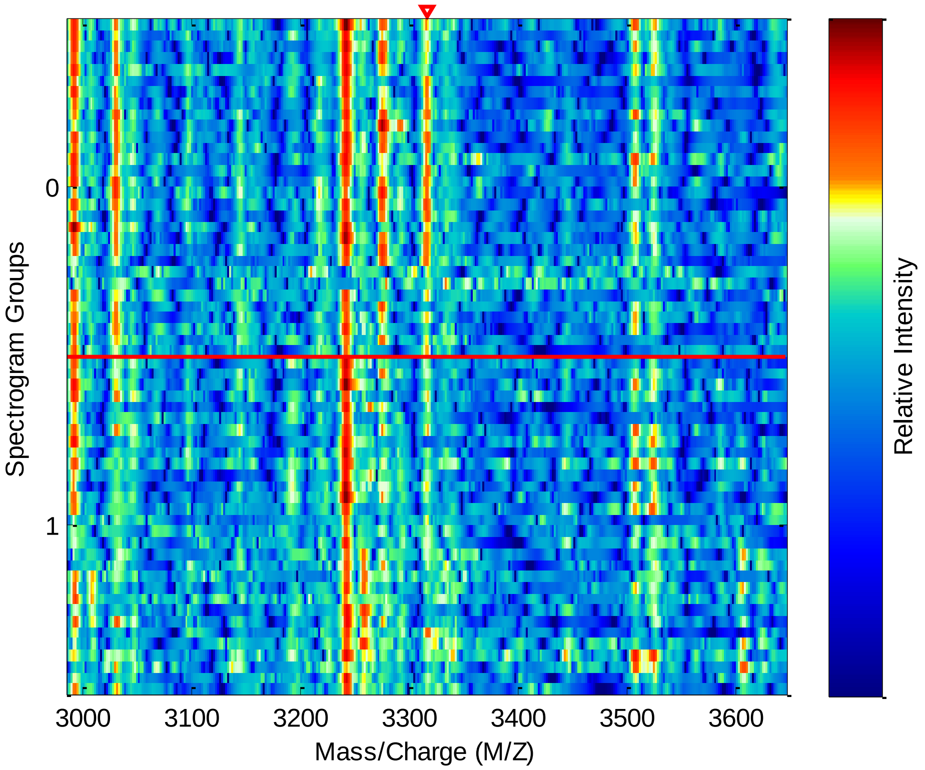

Gel figure: M/Z 3315

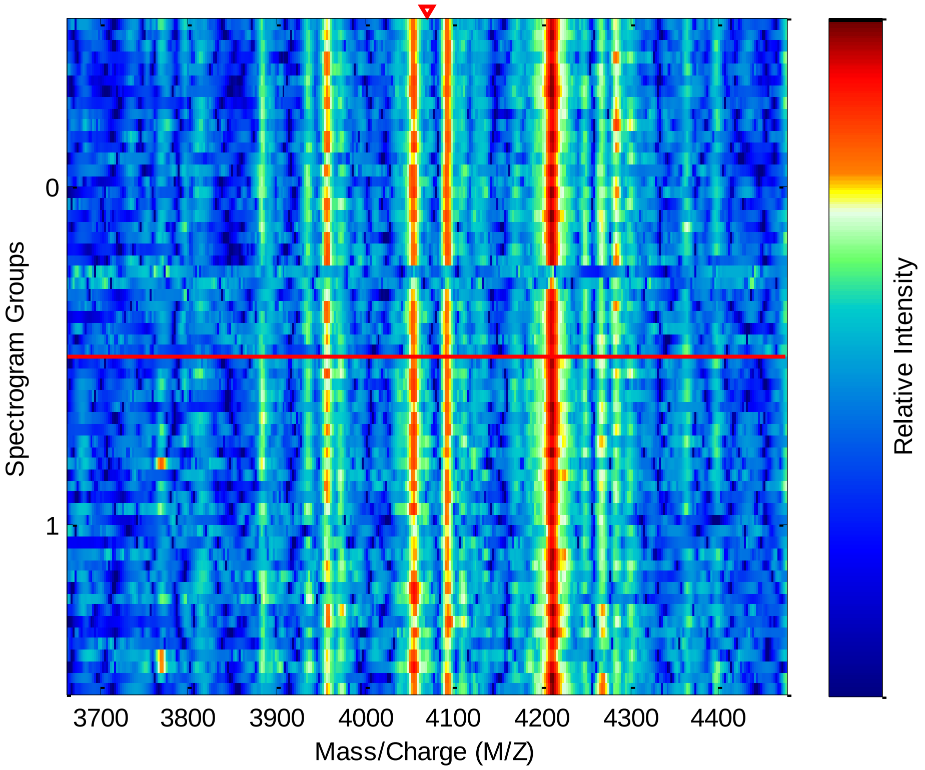

Gel figure: M/Z 4069

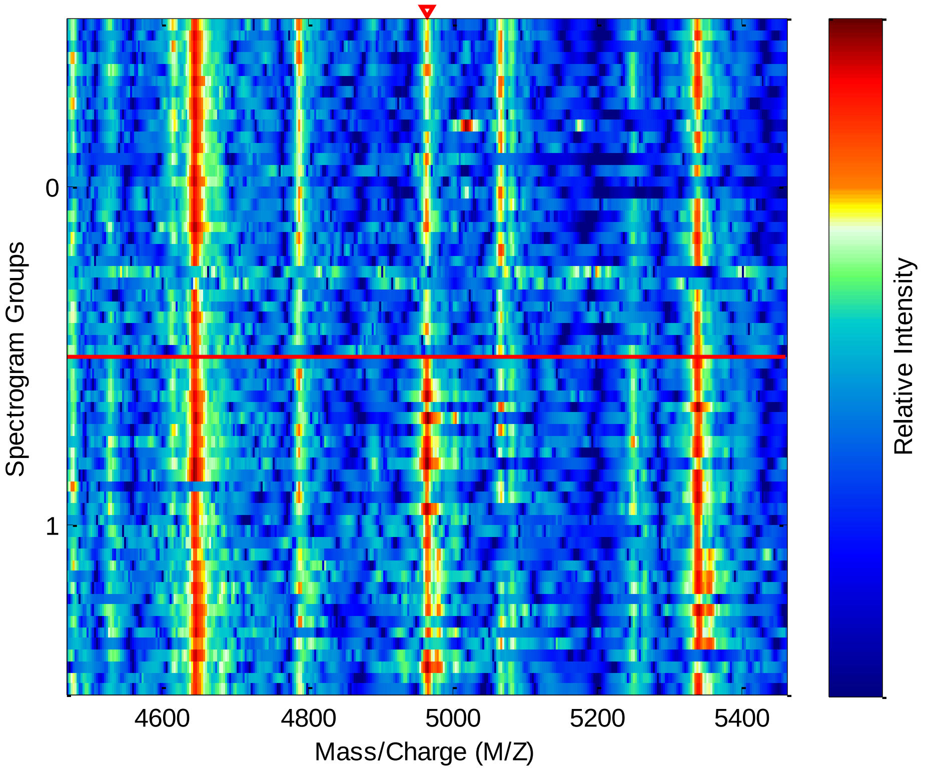

Gel figure: M/Z 4964

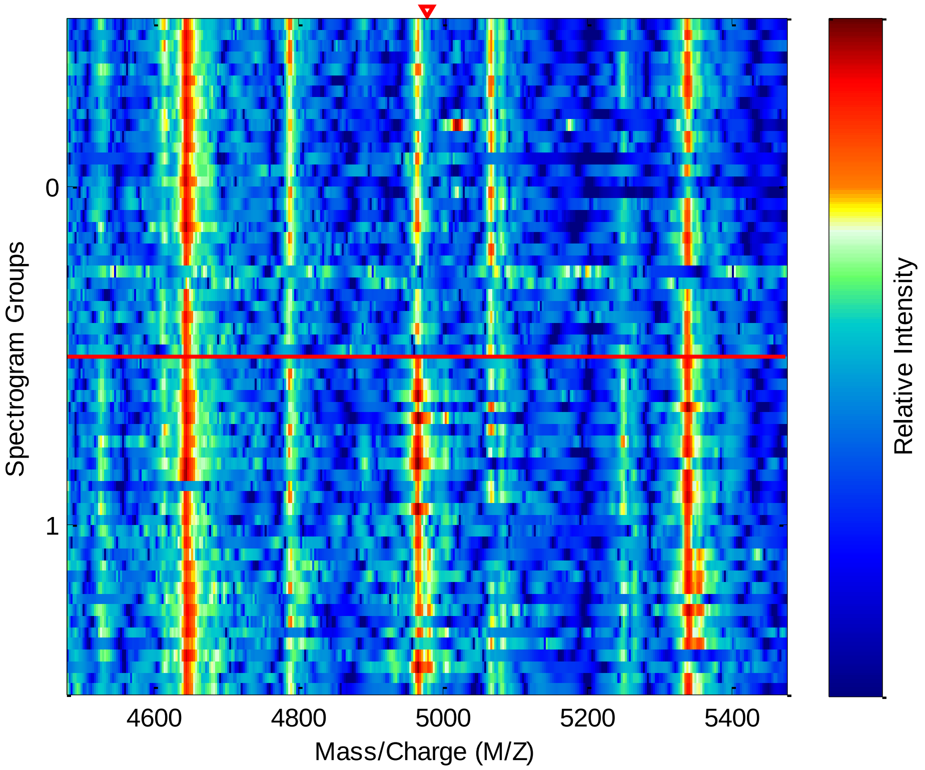

Gel figure: M/Z 4976

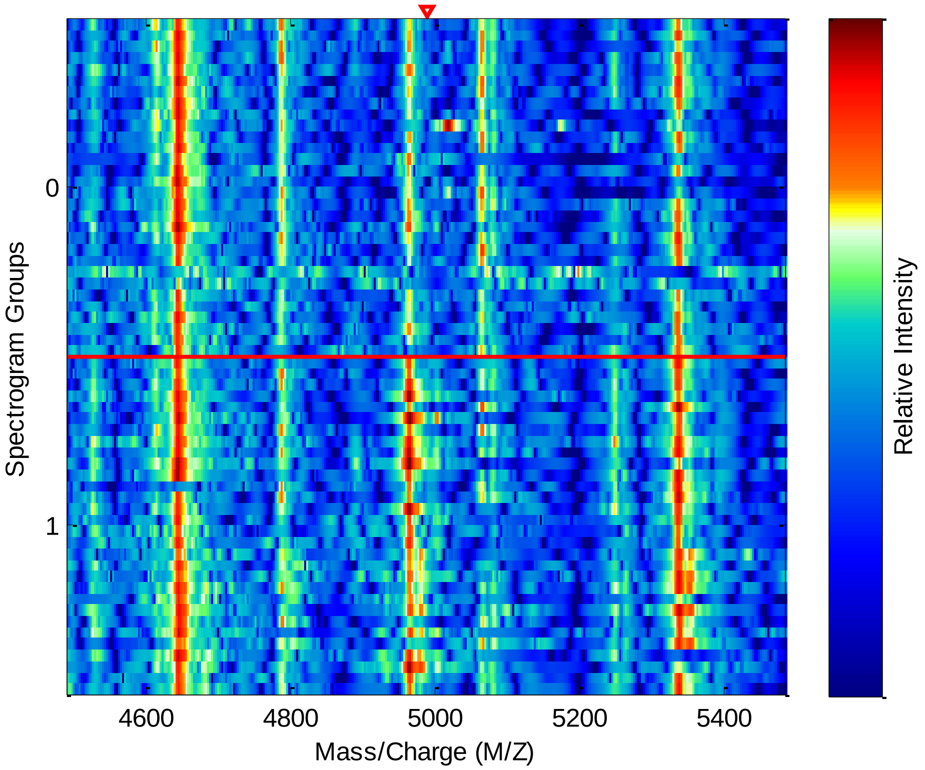

Gel figure: M/Z 4988

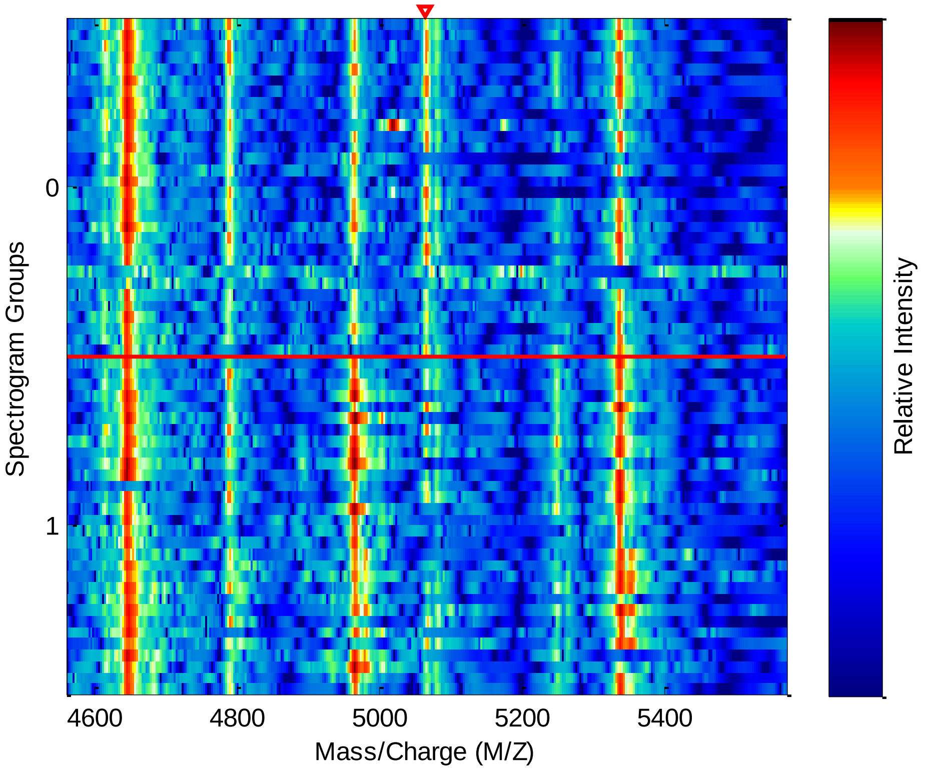

Gel figure: M/Z 5065

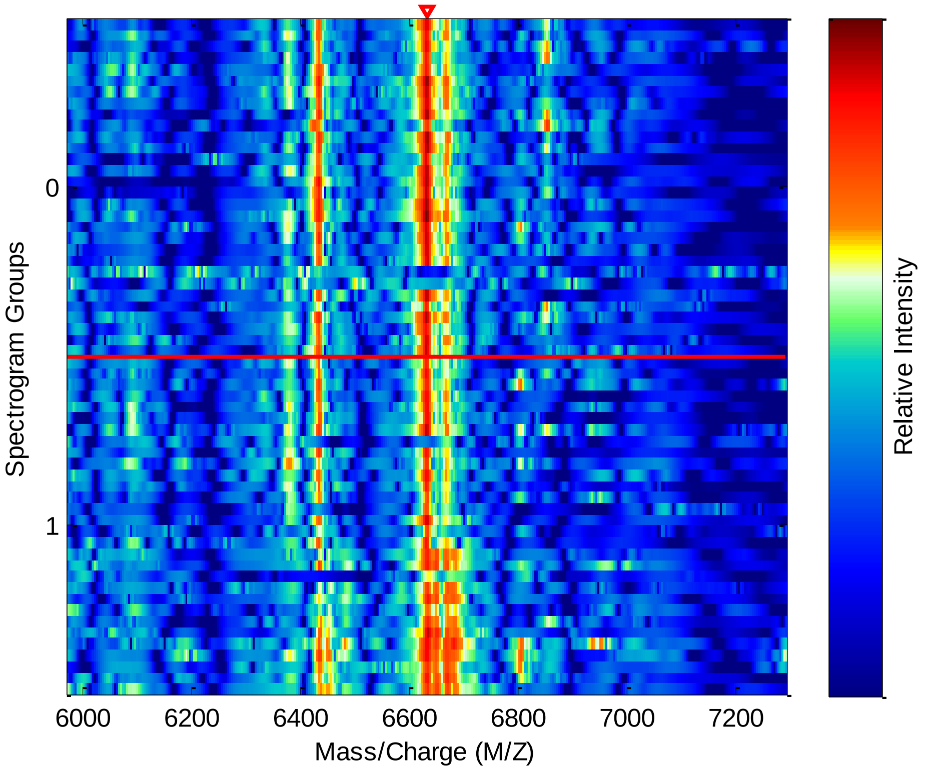

Gel figure: M/Z 6630

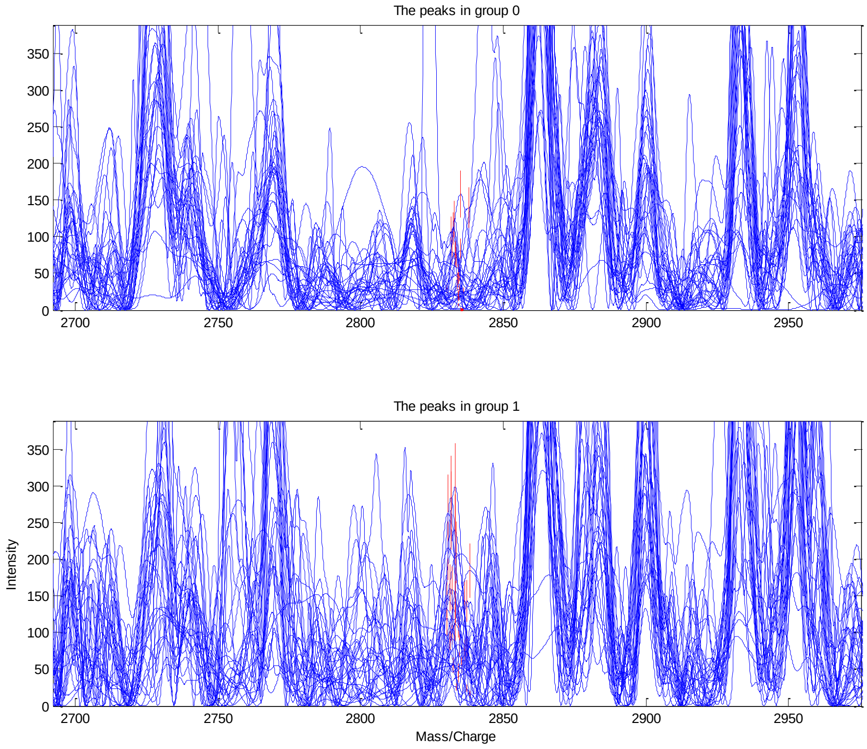

Peak figure: M/Z 2833

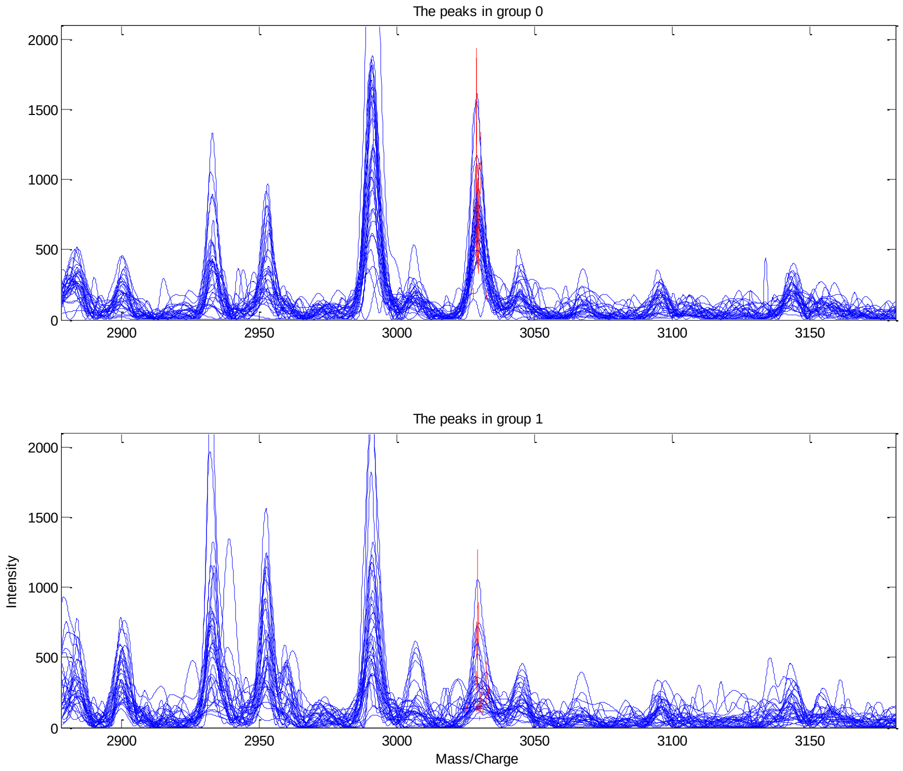

Peak figure: M/Z 3029

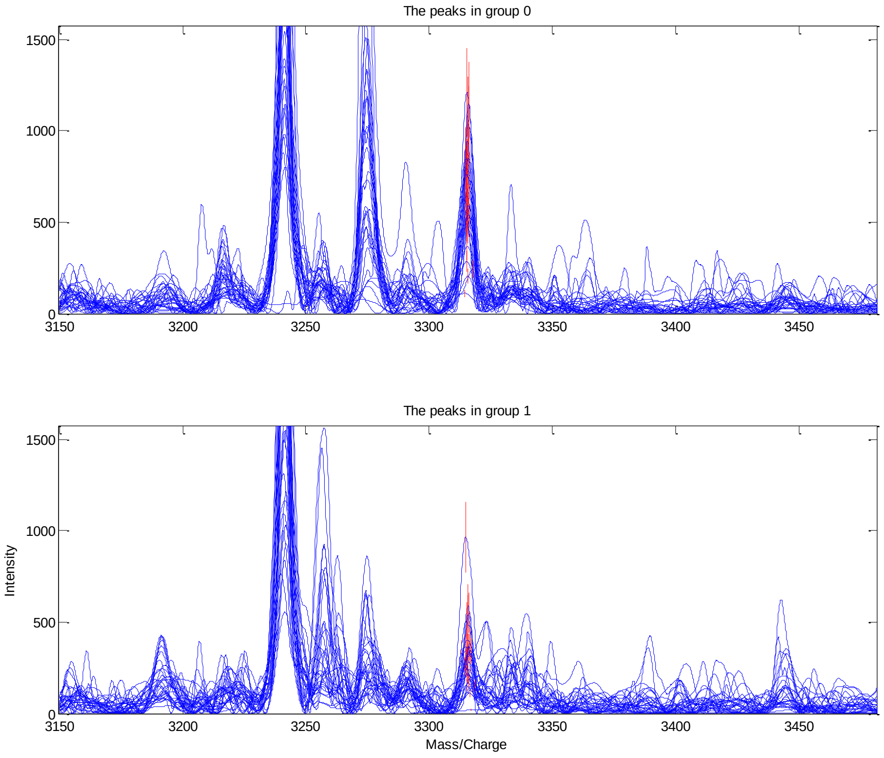

Peak figure: M/Z 3115

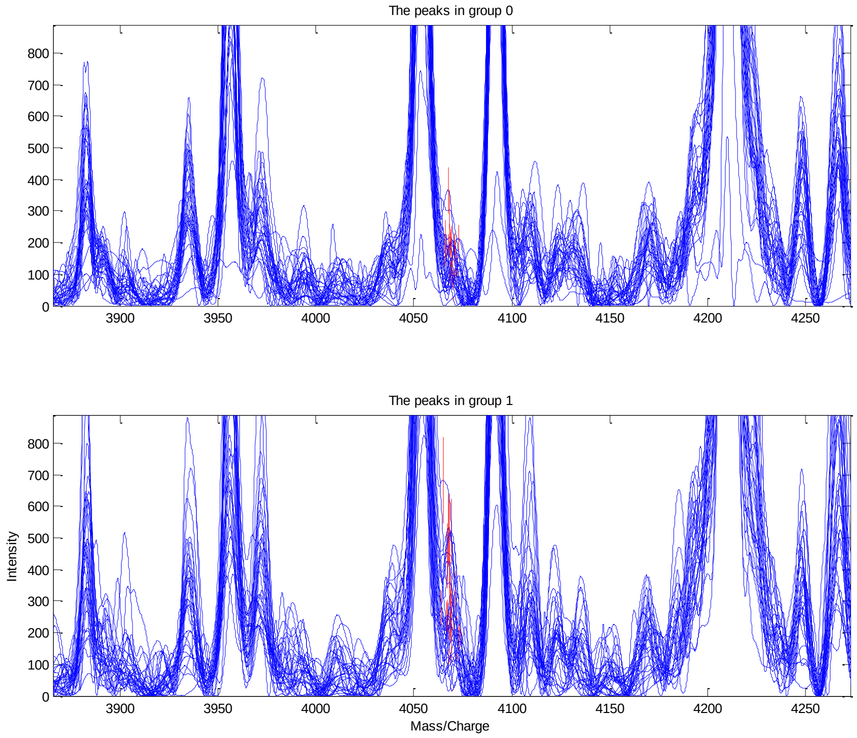

Peak figure: M/Z 4069

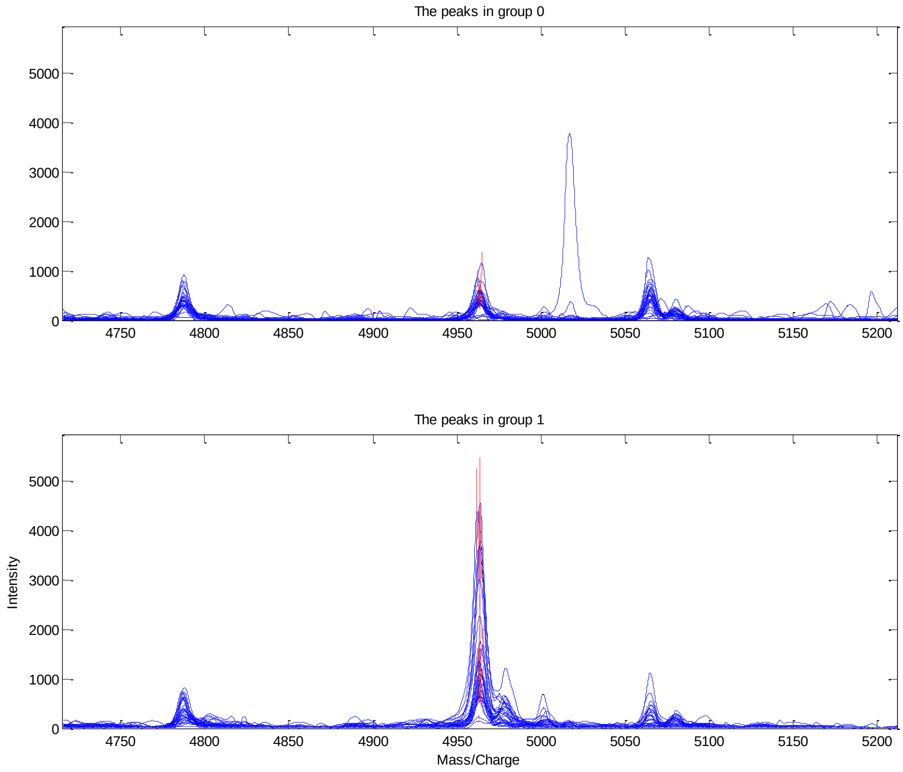

Peak figure: M/Z 4964

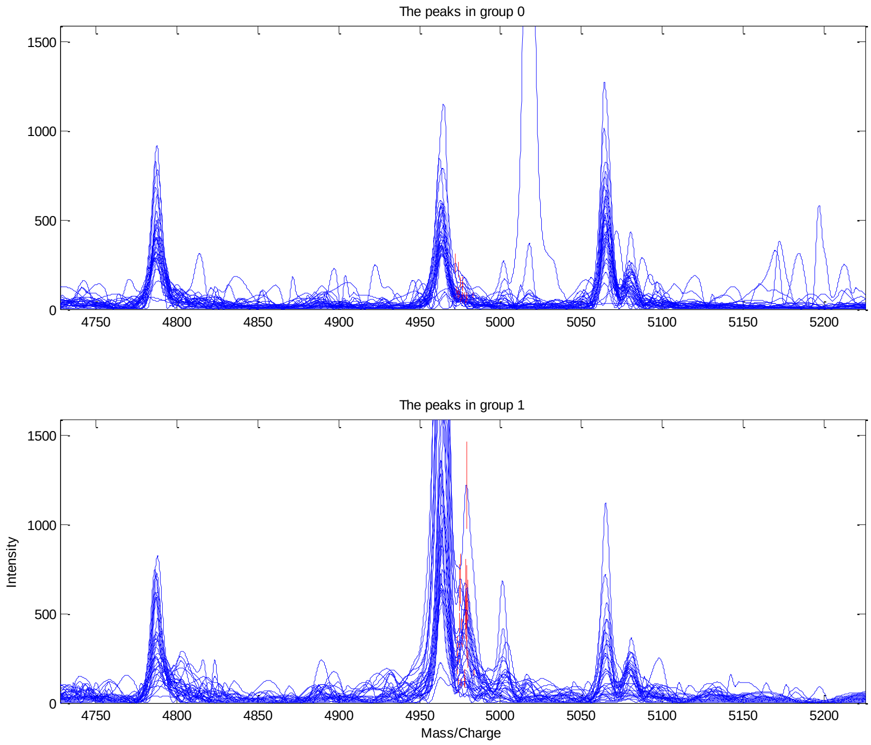

Peak figure: M/Z 4976

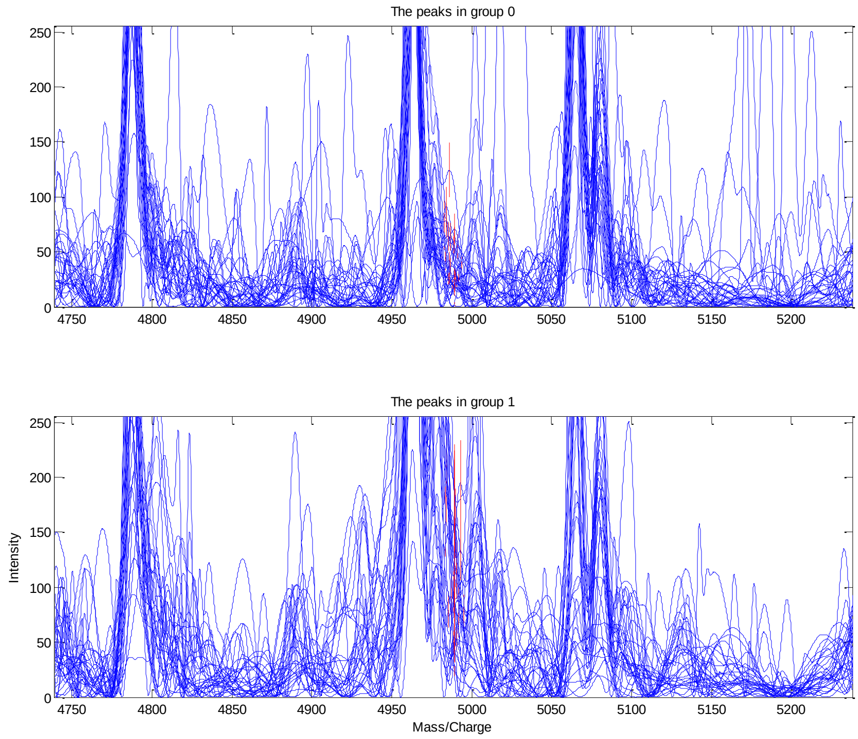

Peak figure: M/Z 4988

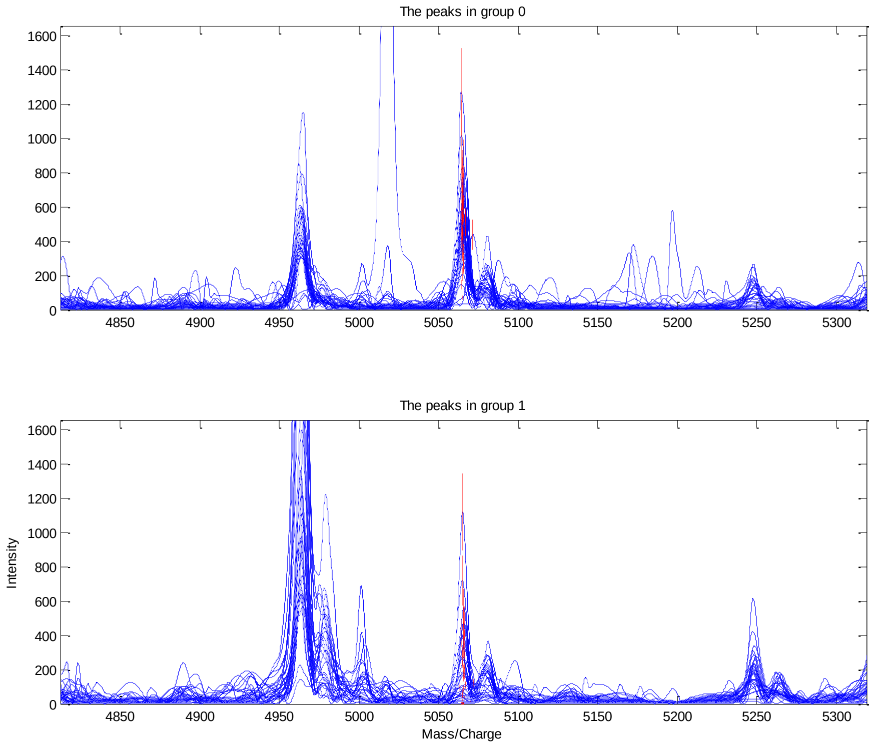

Peak figure: M/Z 5065

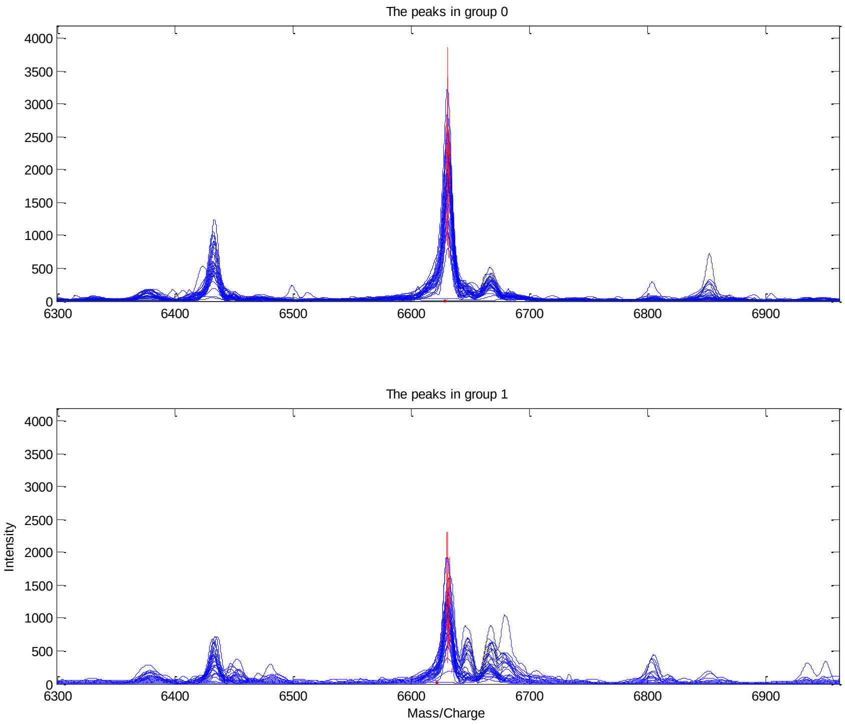

Peak figure: M/Z 6630

User Name zy2016 Peaks filtering factor Wilcoxon
Repeat Sample 0 Small Size Sample No
Test sample percent 0.3 Batch Analyze 1
Wavelet threshold 100 Smoothing window 100
Calibration coefficient (%)0.03 Minimal M/Z (%) 1000
Cluster factor (%) 0.003 Minimal peak threshold (%) 0.1
Excluded spectra threshold (%) 0.2 Minimal sinal/noise ratio (%) 2
Minimal intensity 300 P value or Number of peaks 10
Algorithm 1 Evaluation 1
GA population 50 GA generation 20

Training result

	Predicted Class 0	Predicted Class 1	Sum	Accuracy (%)	Error(%)
Class 0	30	0	30	100	0
Class 1	0	30	30	100	0

Validation result

	Predicted Class 0	Predicted Class 1	Sum	Accuracy (%)	Error (%)
Class 0	29	1	30	96.6667	3.3333
Class 1	1	29	30	96.6667	3.3333

selected markers is
4964.719 3029.7033 2833.9187 2900.1248

Biomarker M/Z
4976.5679 4964.719 3029.7033 4988.552 3315.7248
2833.9187 6630.6521 4069.1778 2900.1248 5065.4939

P value or Weight
1.3111e-008 7.695e-008 7.043e-007 8.1975e-007 3.8349e-006
1.0188e-005 1.5292e-005 2.7726e-005 3.5923e-005 3.5923e-005

Mean in Class0
91.5586 446.2517 747.9188 42.3675 666.6498
50.7423 1746.3756 160.363 207.4617 505.9153

Mean in Class1
369.9864 1585.3402 321.625 108.3447 325.6311
135.6622 1019.3287 299.7414 399.2346 232.4073

Std in Class0
49.6683 225.4799 327.0184 28.542 275.9018

43.03	721.7627	71.6869	114.6405	262.6943

Std in Class1

252.5572	1255.3035	228.6694	51.7756	180.4535
81.0189	436.1463	151.9196	183.067	251.7712
