# Supplementary material for: Exploration of Serum Proteomic Profiling and Diagnostic Model That Differentiate Crohn's Disease and Intestinal Tuberculosis
Source: PLoS One. 2016 Dec 20;11(12):e0167109. doi: 10.1371/journal.pone.0167109 (PMC5173341; doi:10.1371/journal.pone.0167109)
Supplement: S2 File — The biomarkers principal component scatter plot, the gel figure and peak figure of the ten most differentially expressed peaks and the results by using genetic algorithm combining with SVM. (PDF) [file pone.0167109.s002.pdf]

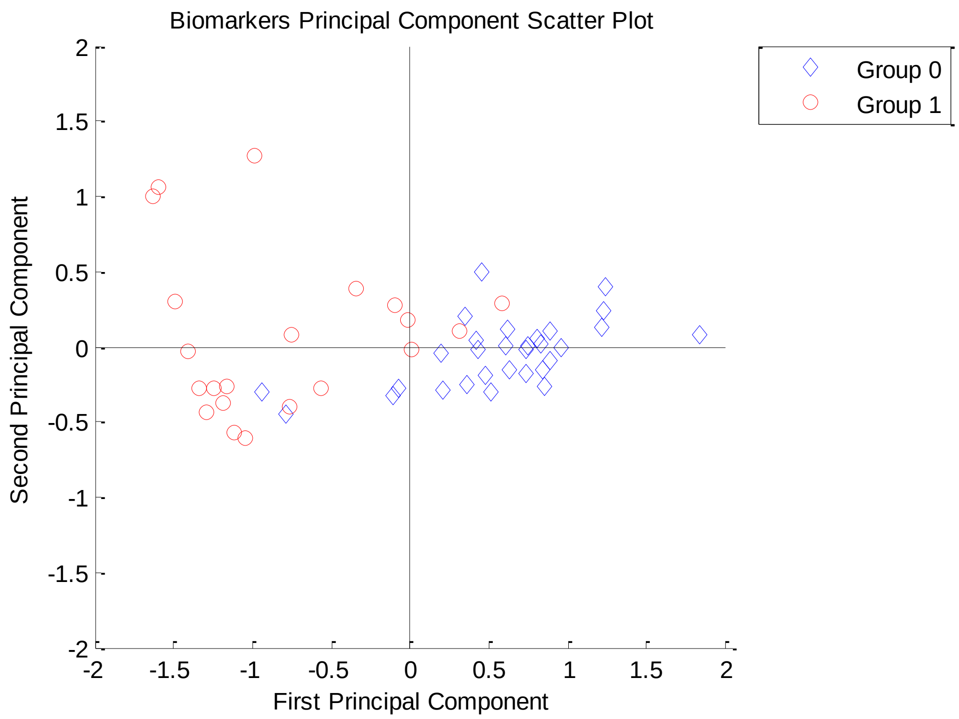

Biomarkers Principal Component Scatter Plot

Group 1: Intestinal tuberculosis patients, group 0: Healthy controls.

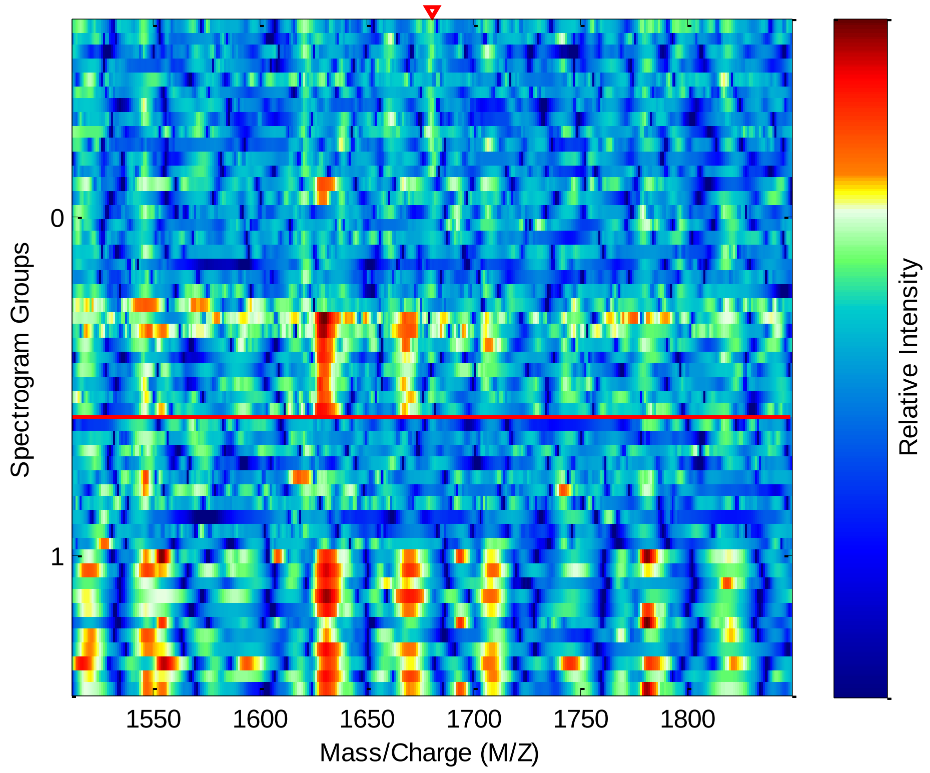

Gel figure: M/Z 1680

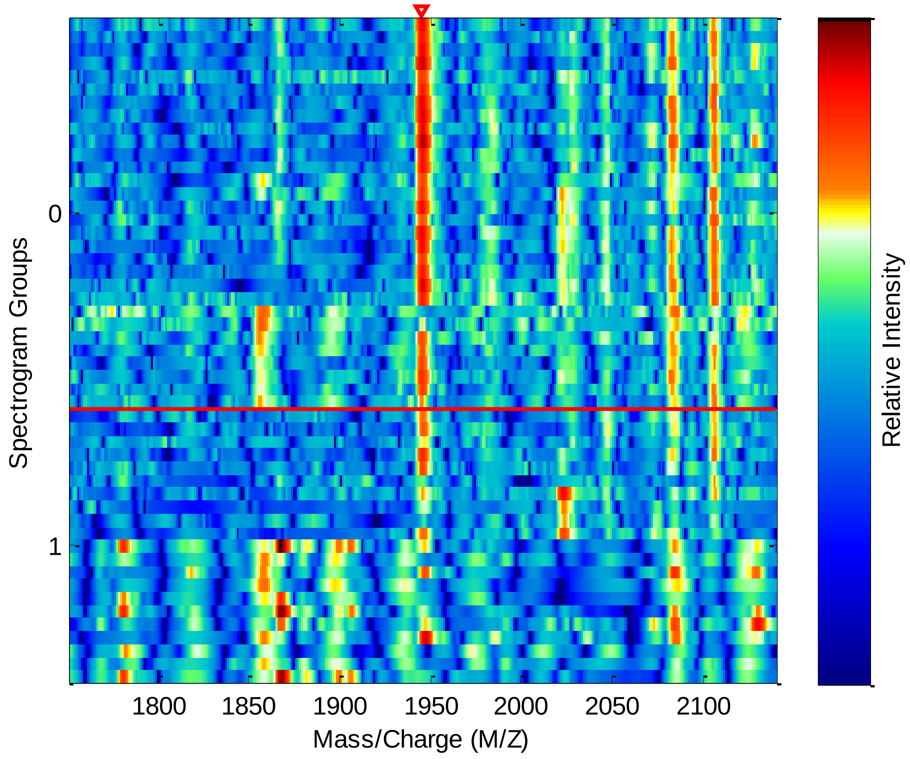

Gel figure: M/Z 1945

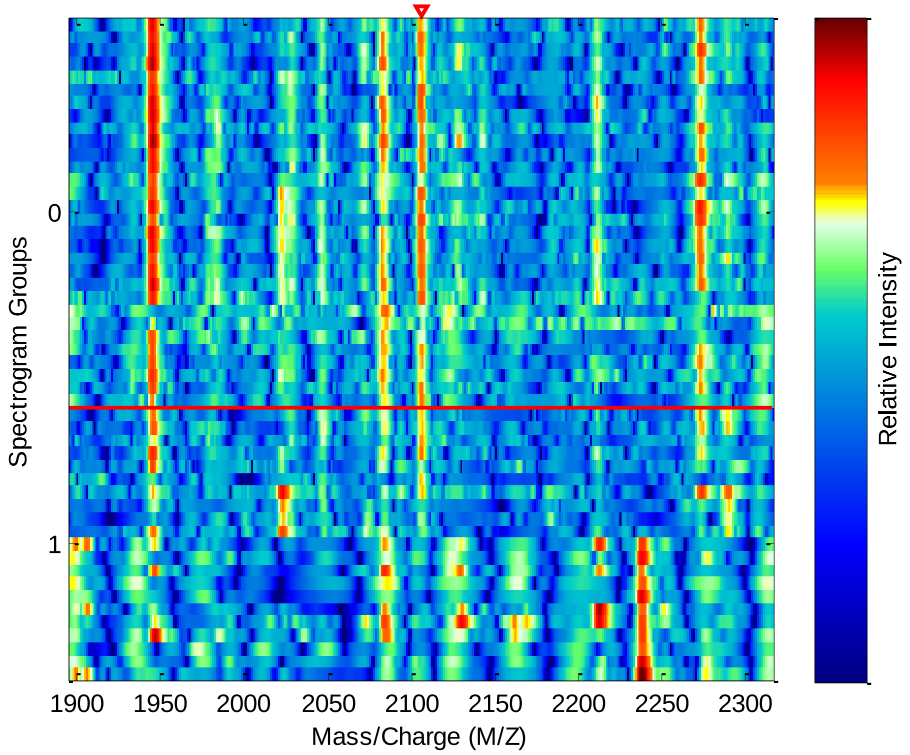

Gel figure: M/Z 2105

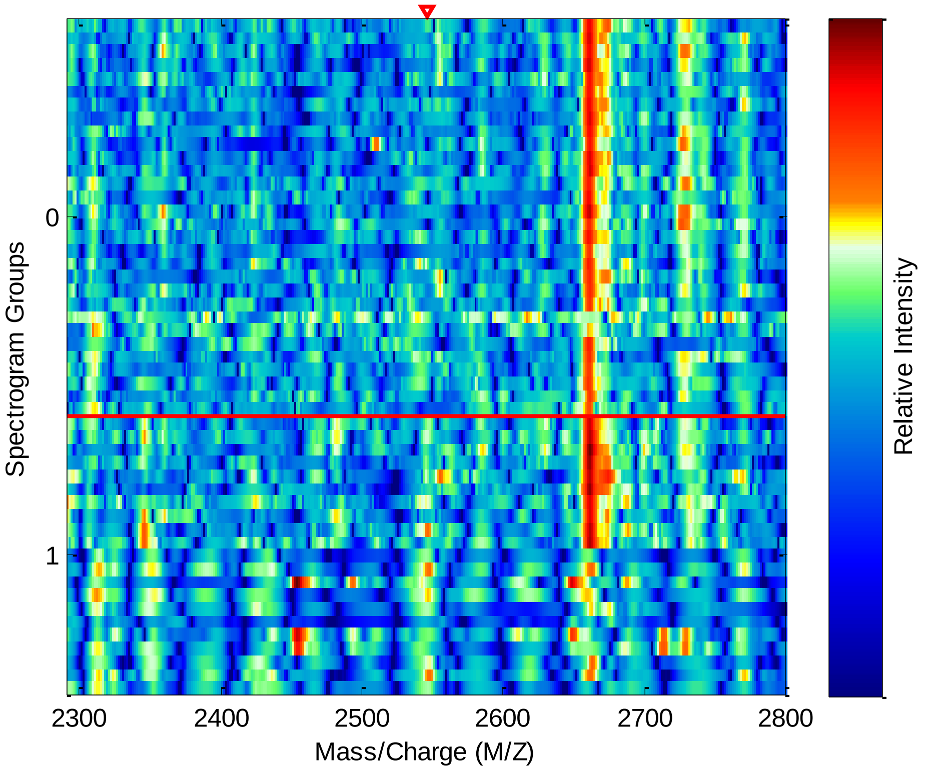

Gel figure: M/Z 2545

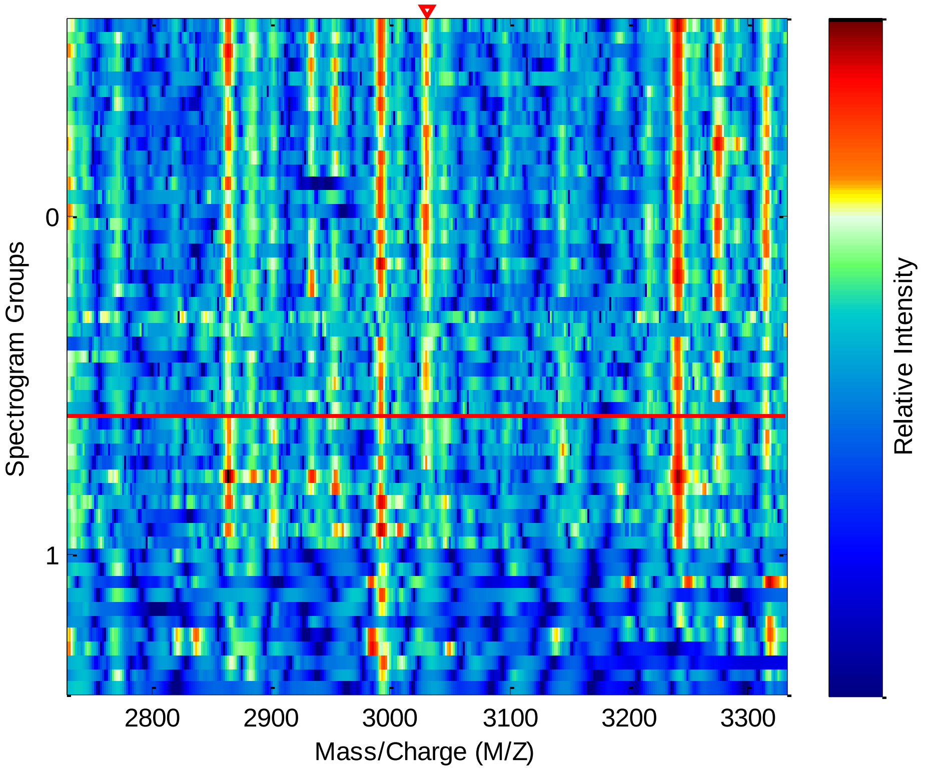

Gel figure: M/Z 3030

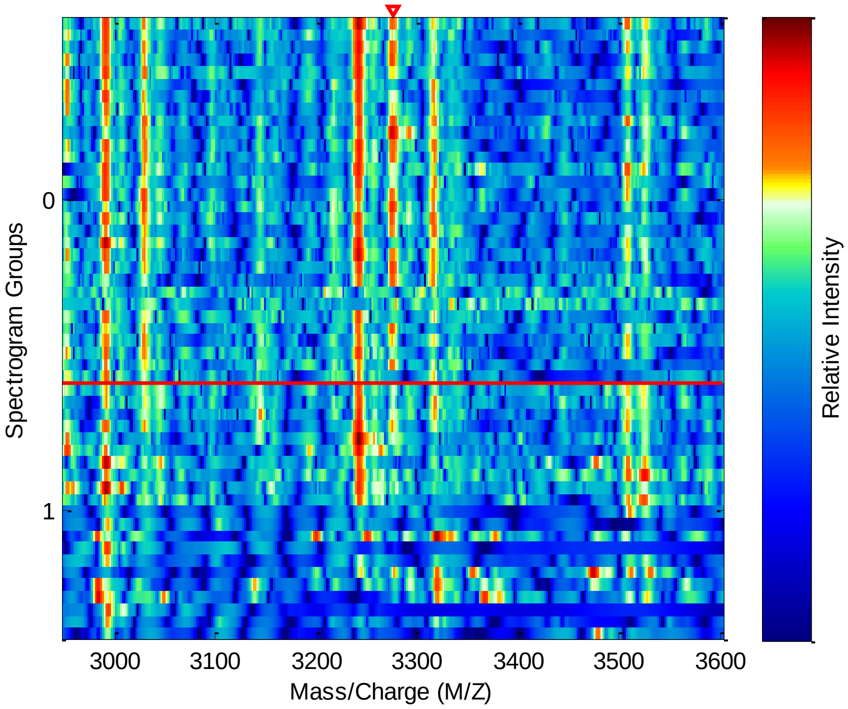

Gel figure: M/Z 3275

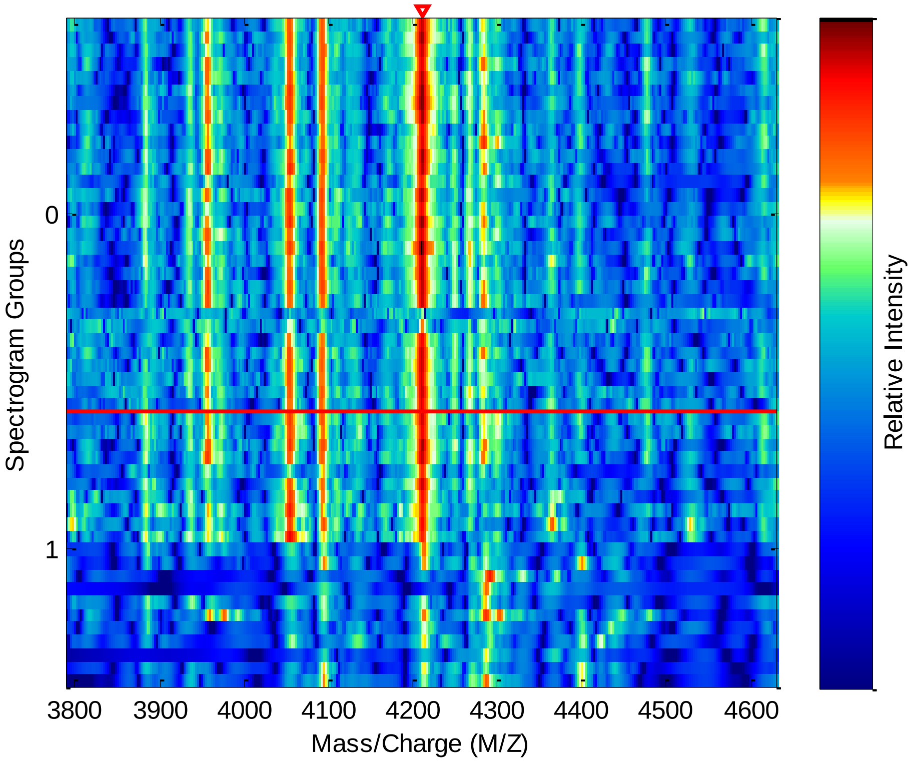

Gel figure: M/Z 4210

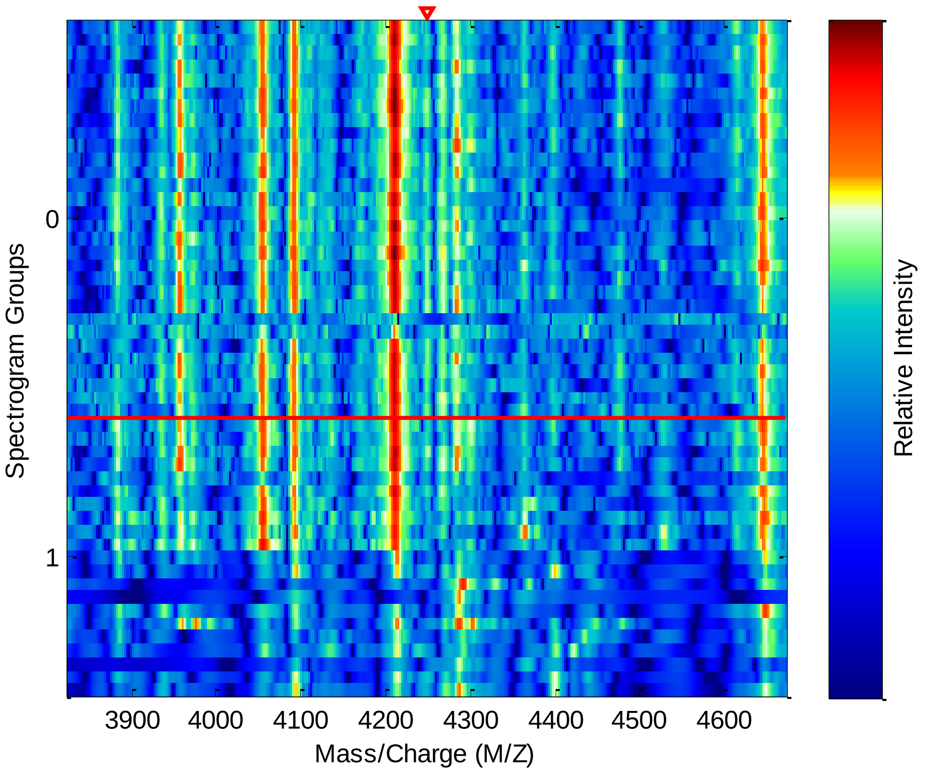

Gel figure: M/Z 4248

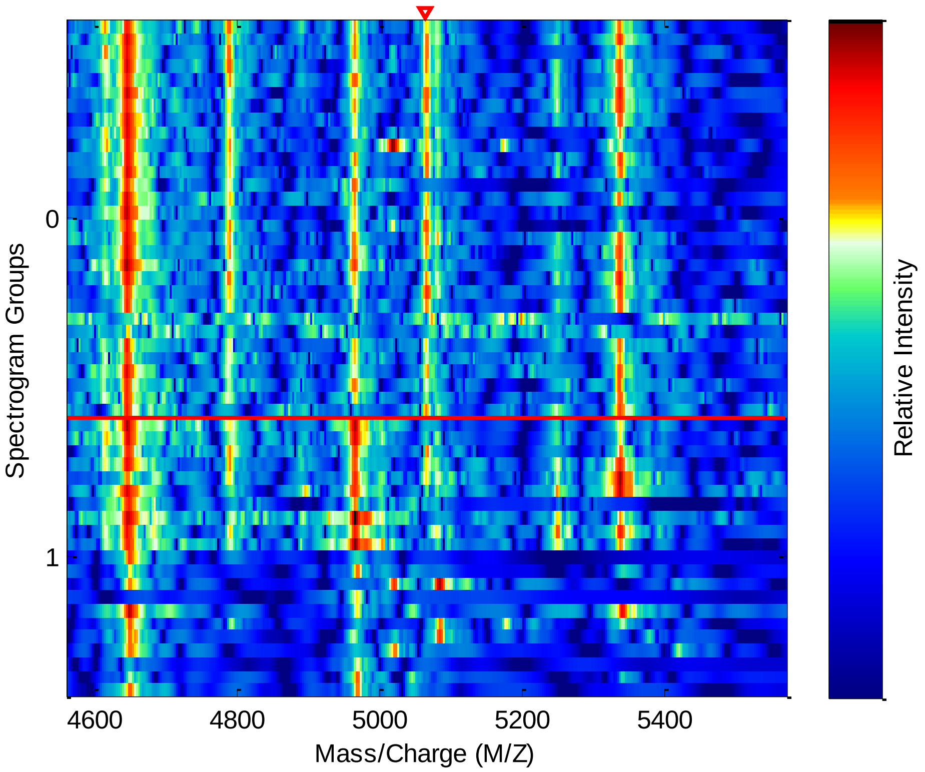

Gel figure: M/Z 5065

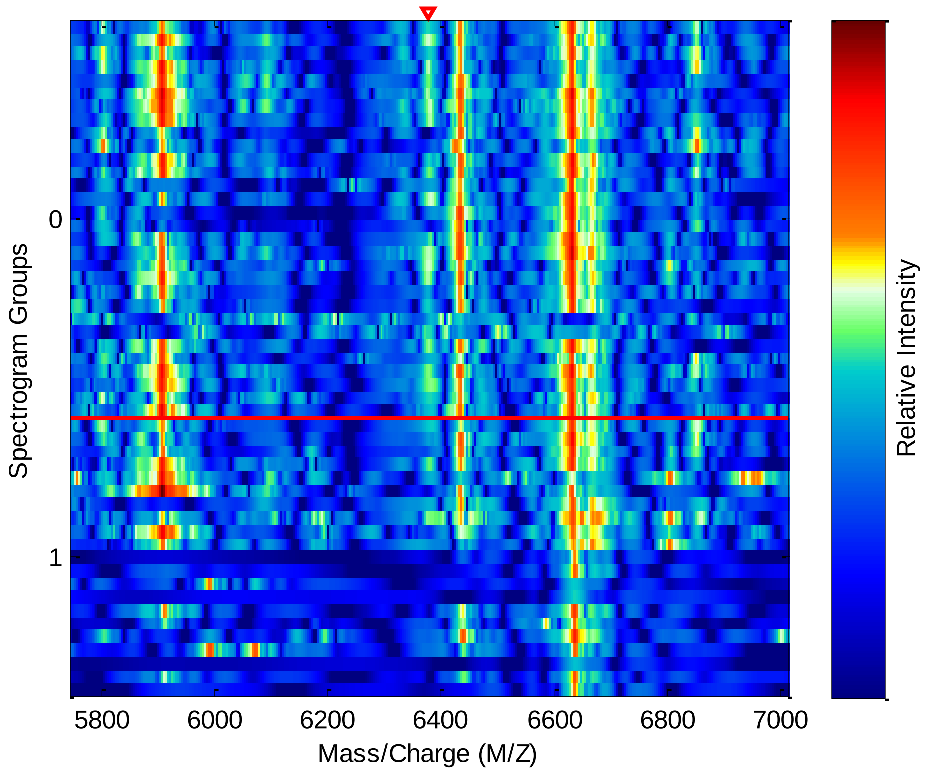

Gel figure: M/Z 6379

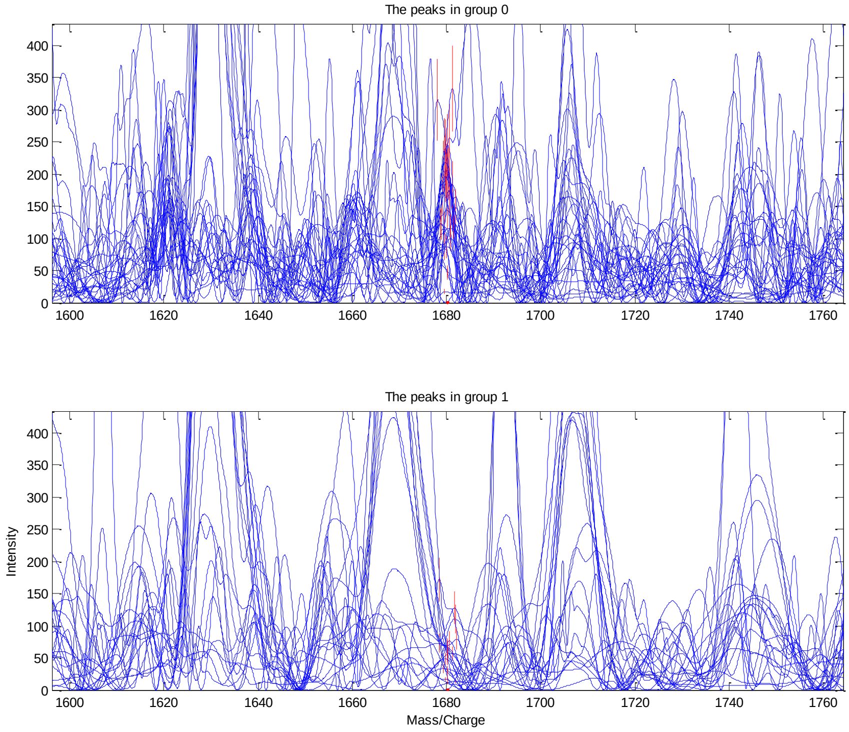

Peak figure: M/Z 1680

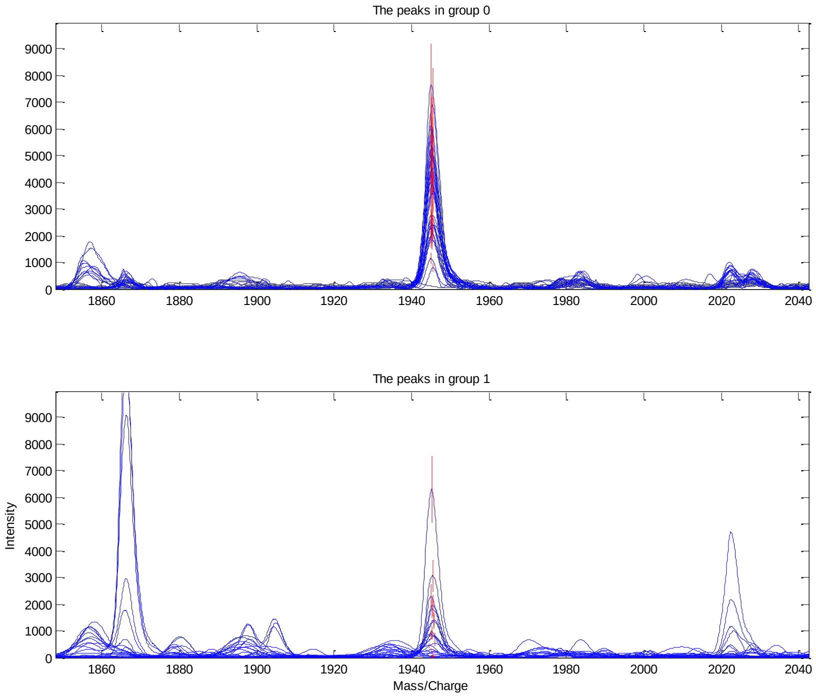

Peak figure: M/Z 1945

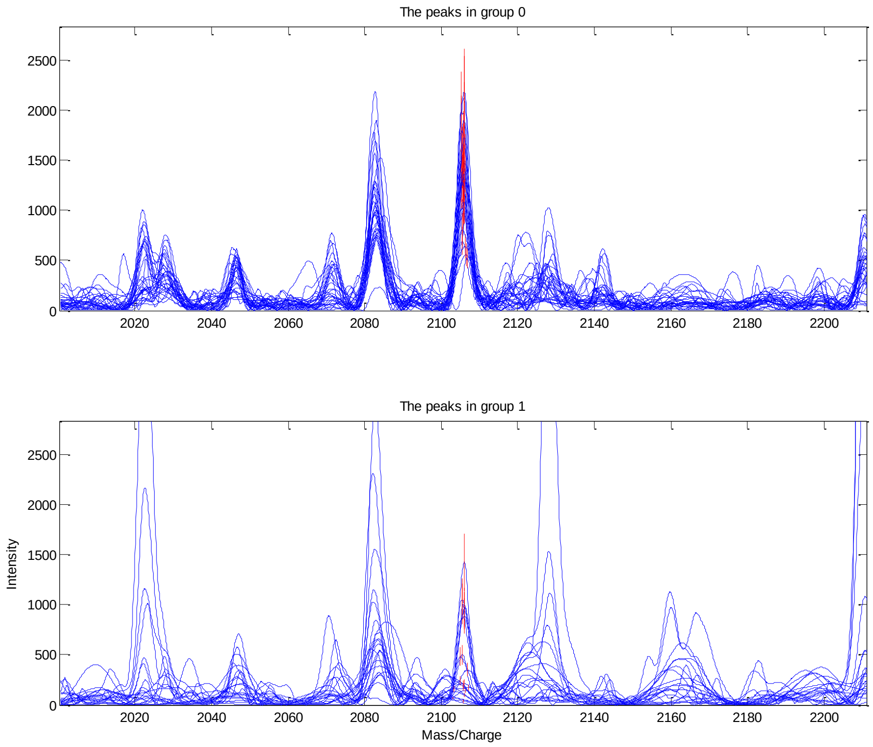

Peak figure: M/Z 2105

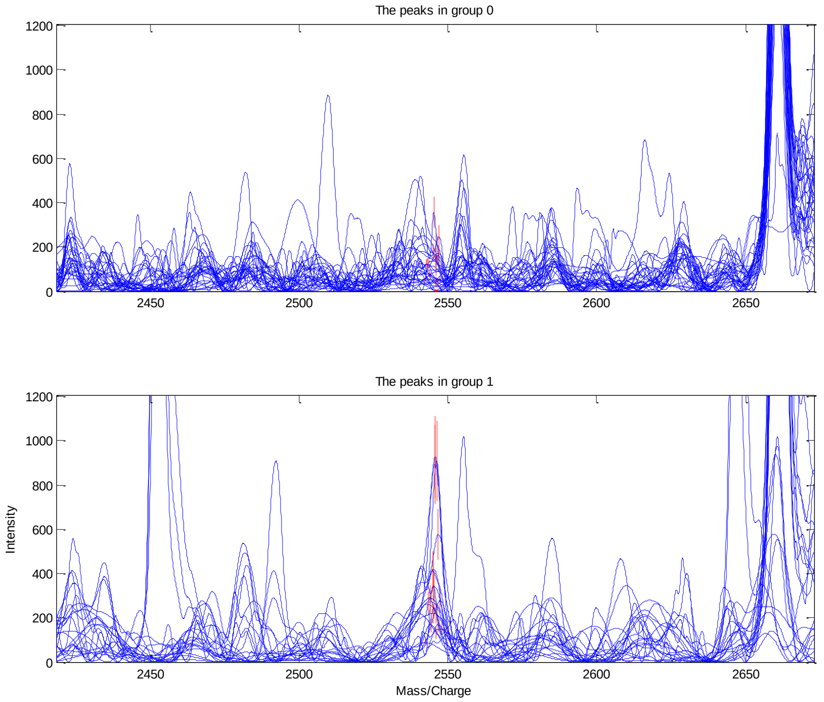

Peak figure: M/Z 2545

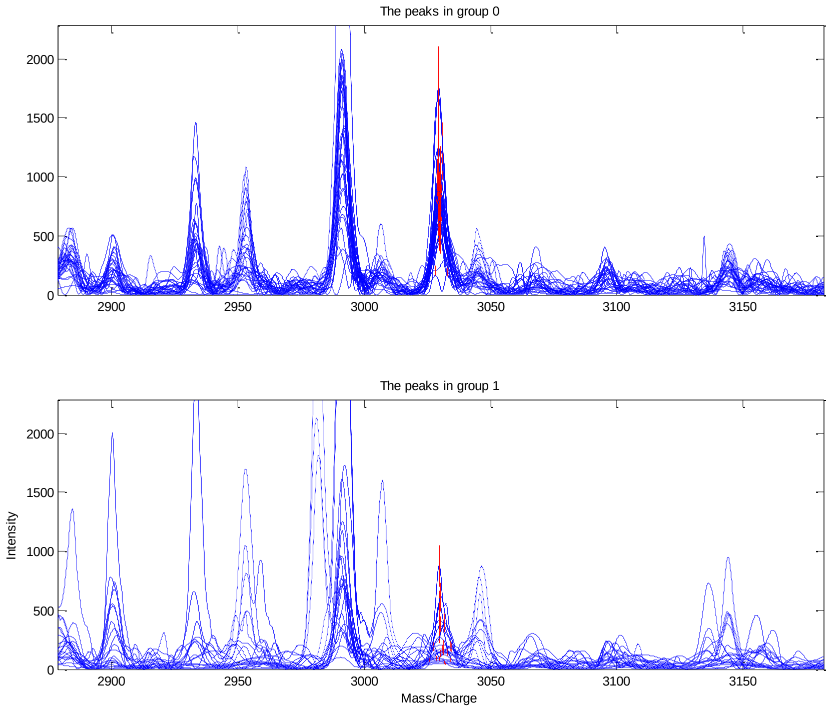

Peak figure: M/Z 3030

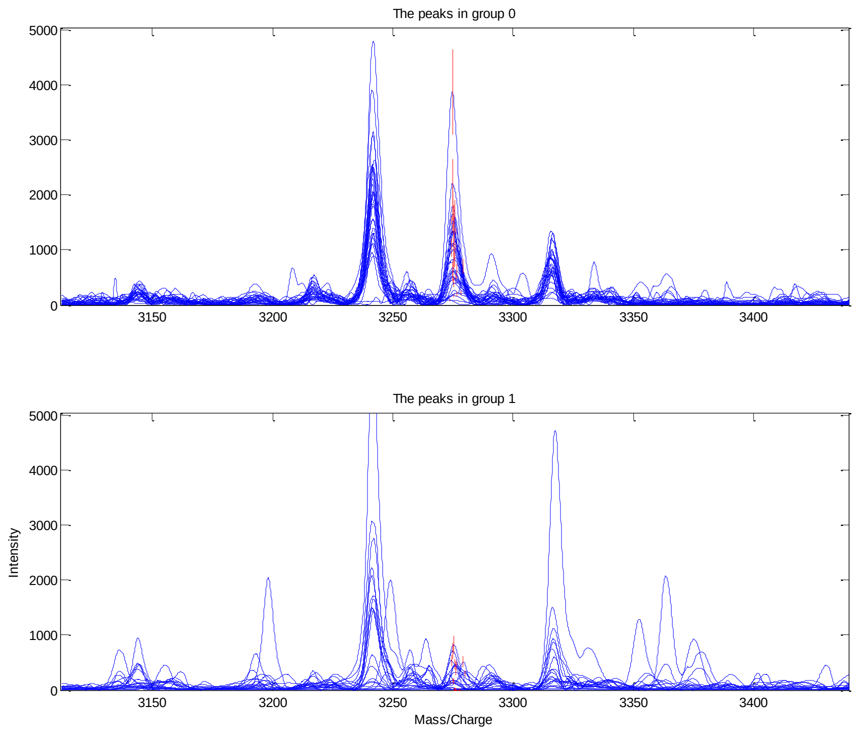

Peak figure: M/Z 3275

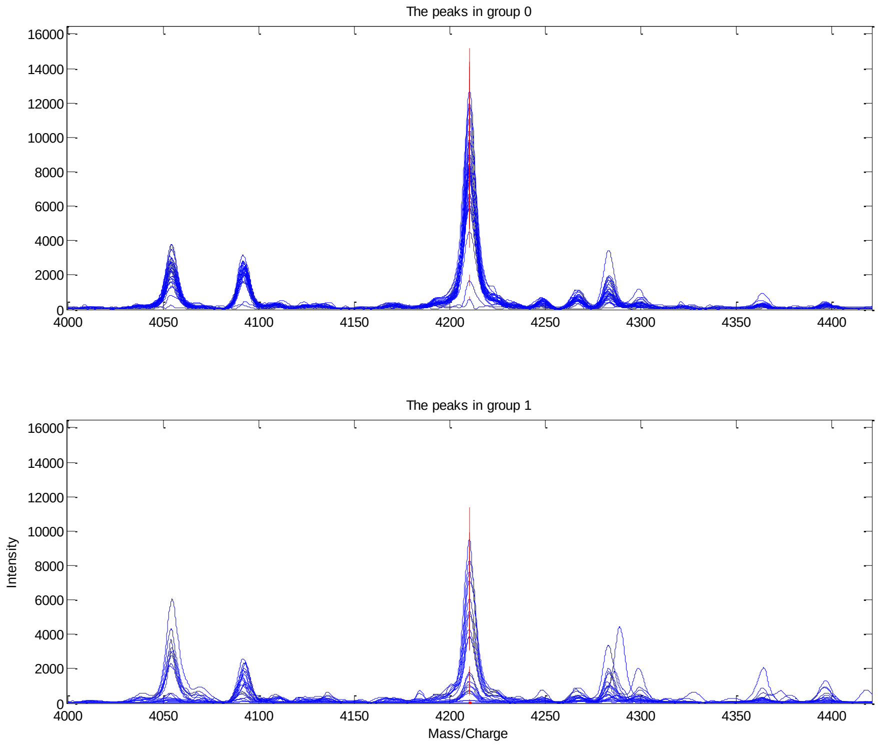

Peak figure: M/Z 4210

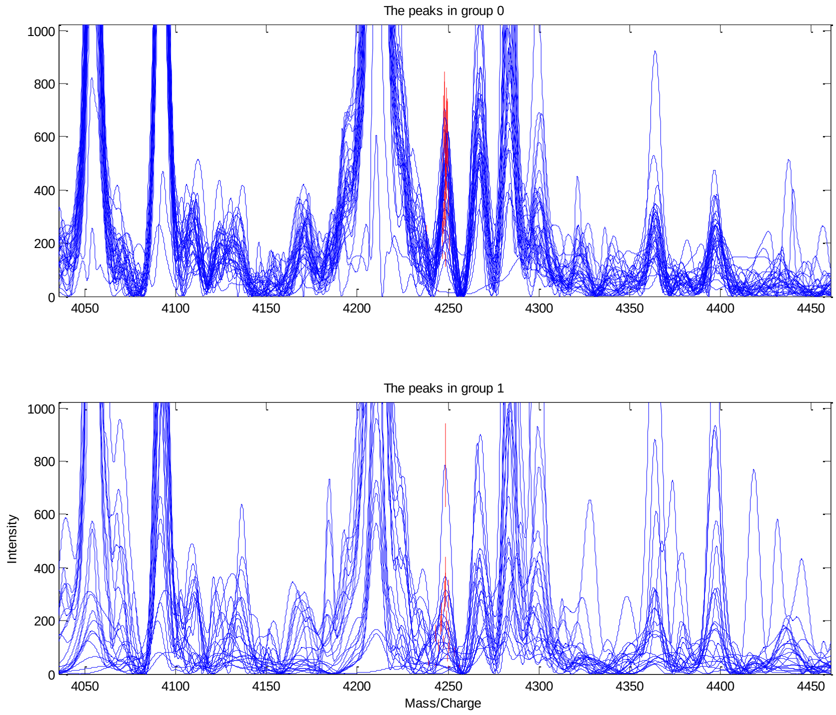

Peak figure: M/Z 4248

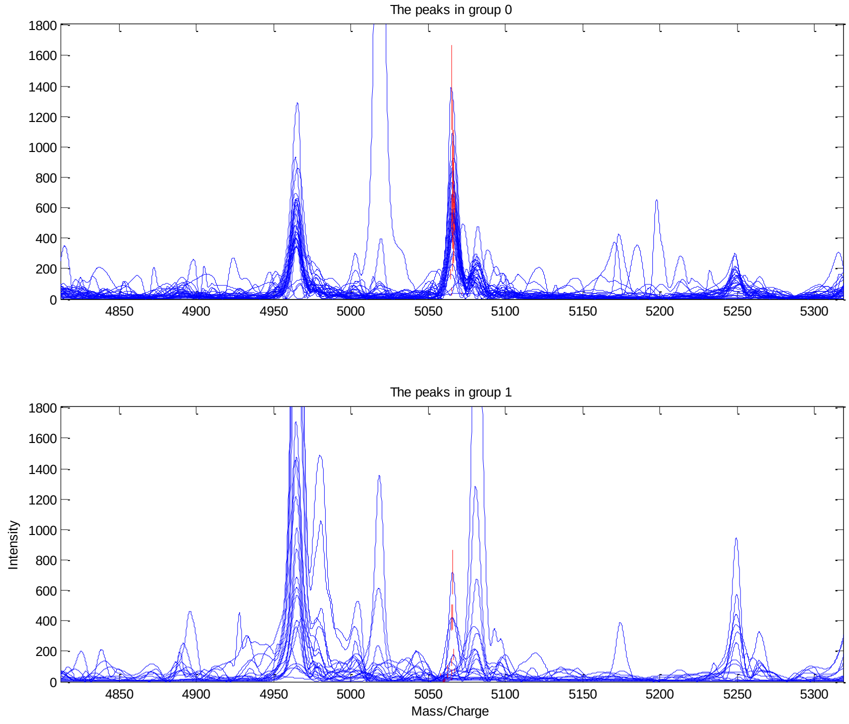

Peak figure: M/Z 5065

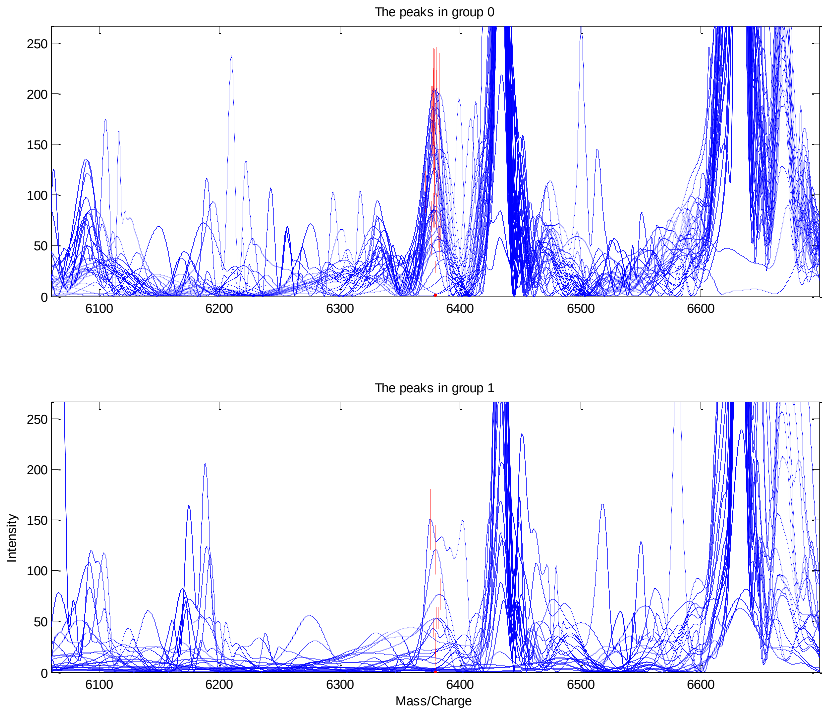

Peak figure: M/Z 6379

User Name zy2016 Peaks filtering factor Wilcoxon

Repeat Sample 0 Small Size Sample No

Test sample percent 0.3 Batch Analyze 1

Wavelet threshold 100 Smoothing window 100

Calibration coefficient (%)0.03 Minimal M/Z (%) 1000

Cluster factor (%) 0.003 Minimal peak threshold (%) 0.1

Excluded spectra threshold (%) 0.2 Minimal sinal/noise ratio (%) 2

Minimal intensity 300 P value or Number of peaks 10

Algorithm 1 Evaluation 1

GA population 50 GA generation 20

Training result

	Predicted Class 0	Predicted Class 1	Sum	Accuracy (%)	Error (%)
Class 0	30	0	30	100	0
Class 1	1	20	21	95.2381	4.7619

Validation result

	Predicted Class 0	Predicted Class 1	Sum	Accuracy (%)	Error(%)
Class 0	28	2	30	93.3333	6.6667
Class 1	1	20	21	95.2381	4.7619

selected markers is
3030.248 2105.7772 2545.6704 4210.5168
Biomarker M/Z
3030.248 2105.7772 2545.6704 5065.0708 1945.2667
1680.2109 3275.7756 4210.5168 6379.0283 4248.0205

P value or Weight
2.0337e-007 3.7467e-007 1.0083e-006 1.9743e-006 4.166e-006
1.0264e-005 1.0264e-005 1.0264e-005 1.2241e-005 2.2423e-005

Mean in Class0
828.0201 1325.0445 100.562 544.6942 3689.9815
162.0247 999.0508 7918.5456 112.5922 419.392

Mean in Class1
254.2988 439.3655 355.371 117.292 1128.5762
56.6174 261.9605 3333.2838 30.197 185.8596

Std in Class0
352.6482 443.4387 81.0572 303.7012 1842.3398
81.3891 774.3984 2663.1699 64.5077 177.5358

Std in Class1
214.6184 425.7776 258.7236 182.4033 1453.1253
41.6849 252.908 3034.131 41.3063 170.3095
