# Supplementary material for: Exploration of Serum Proteomic Profiling and Diagnostic Model That Differentiate Crohn's Disease and Intestinal Tuberculosis
Source: PLoS One. 2016 Dec 20;11(12):e0167109. doi: 10.1371/journal.pone.0167109 (PMC5173341; doi:10.1371/journal.pone.0167109)
Supplement: S3 File — The biomarkers principal component scatter plot, the gel figure and peak figure of the ten most differentially expressed peaks and the results by using genetic algorithm combining with SVM. (PDF) [file pone.0167109.s003.pdf]

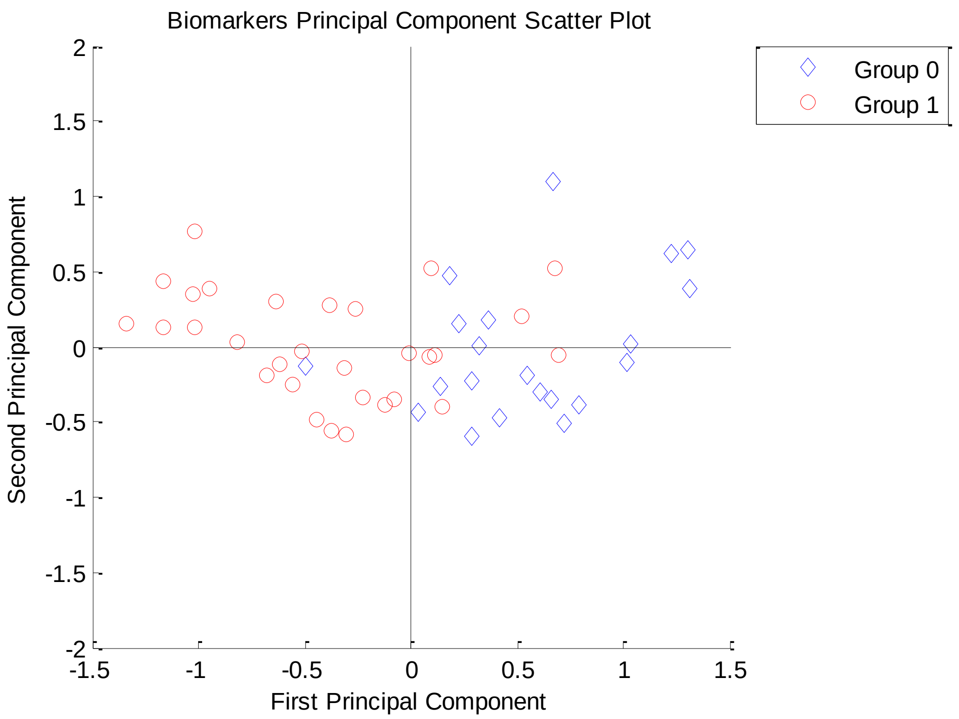

Biomarkers Principal Component Scatter Plot
Group 1: Crohn's disease patients, group 0: Intestinal tuberculosis patients

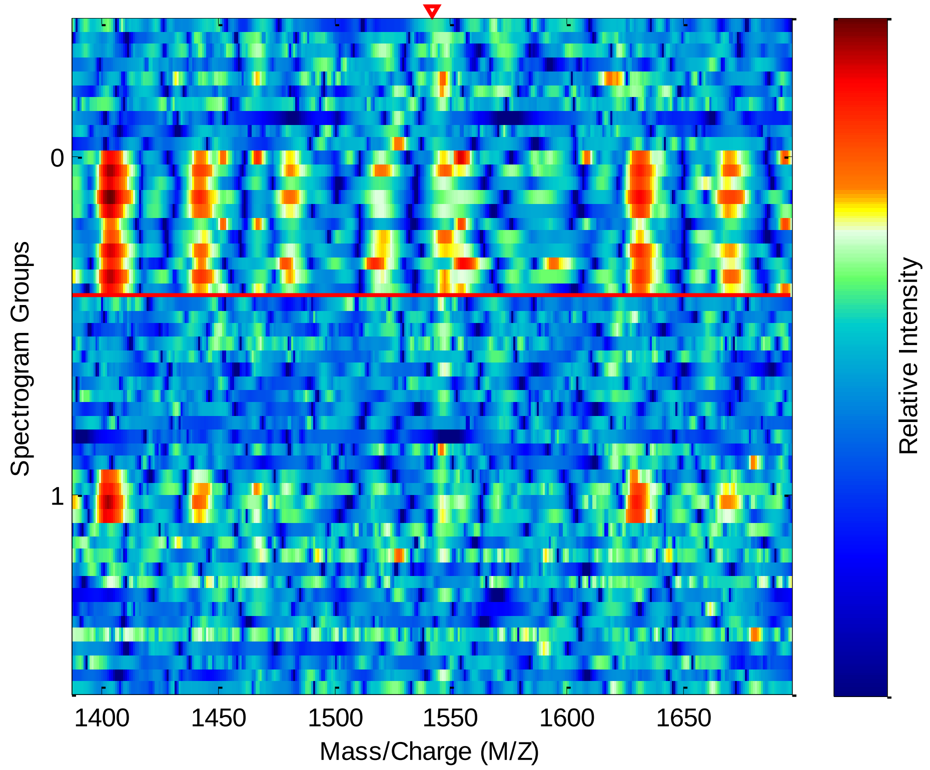

Gel figure: M/Z 1541

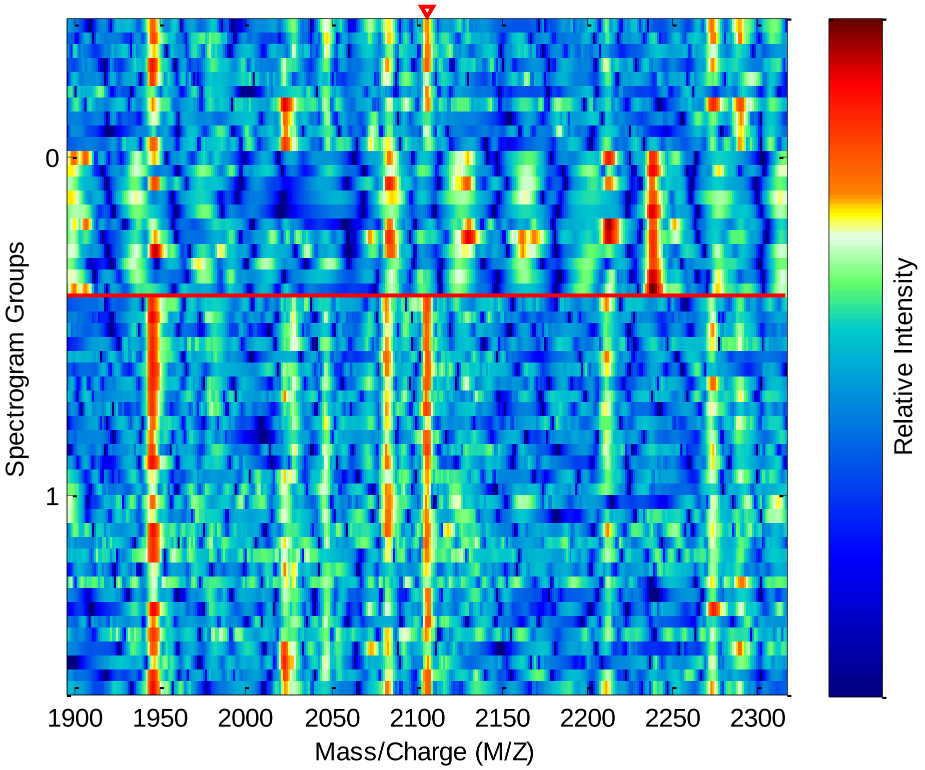

Gel figure: M/Z 2105

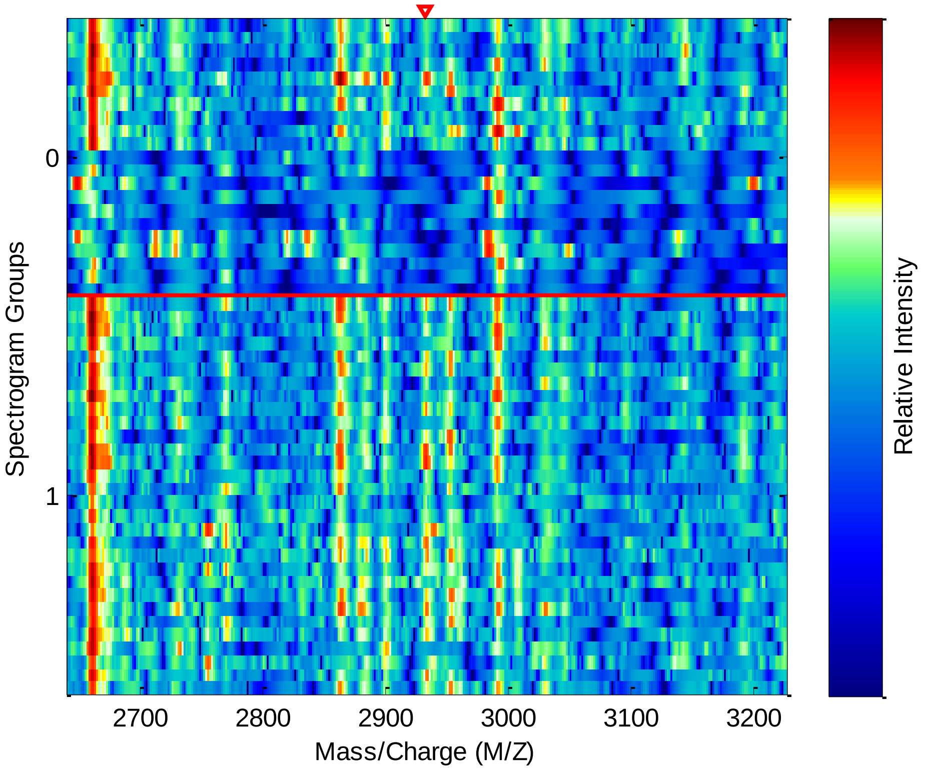

Gel figure: M/Z 2933

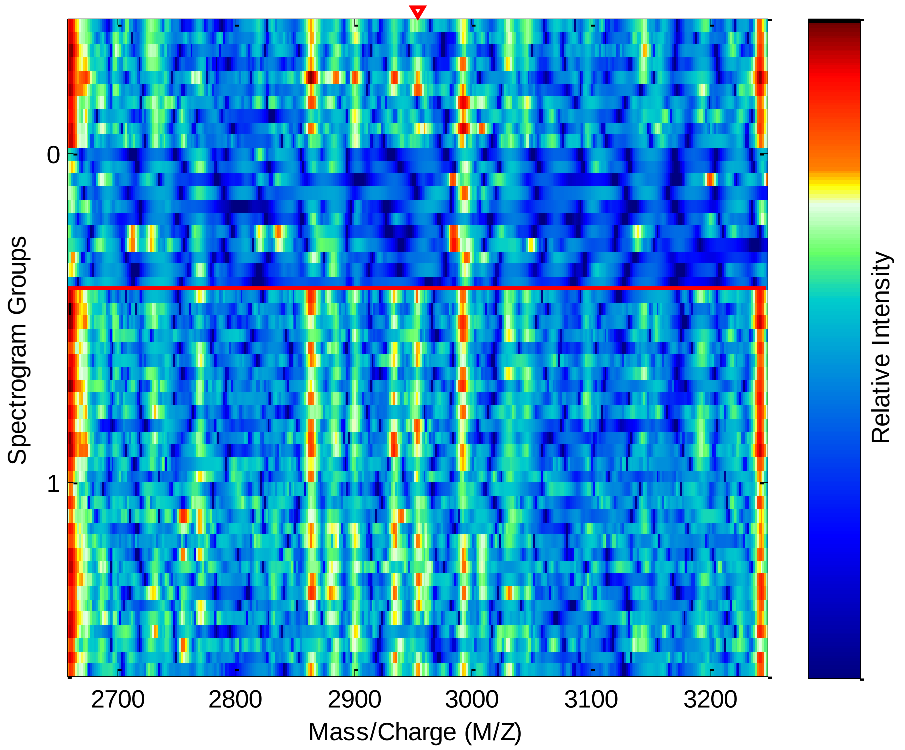

Gel figure: M/Z 2952

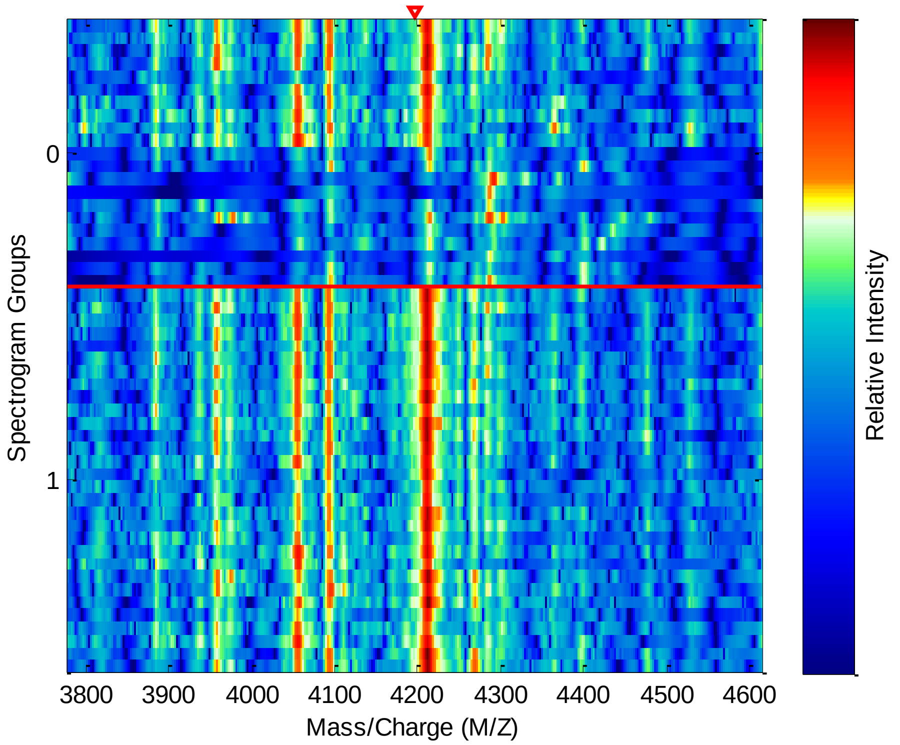

Gel figure: M/Z 4195

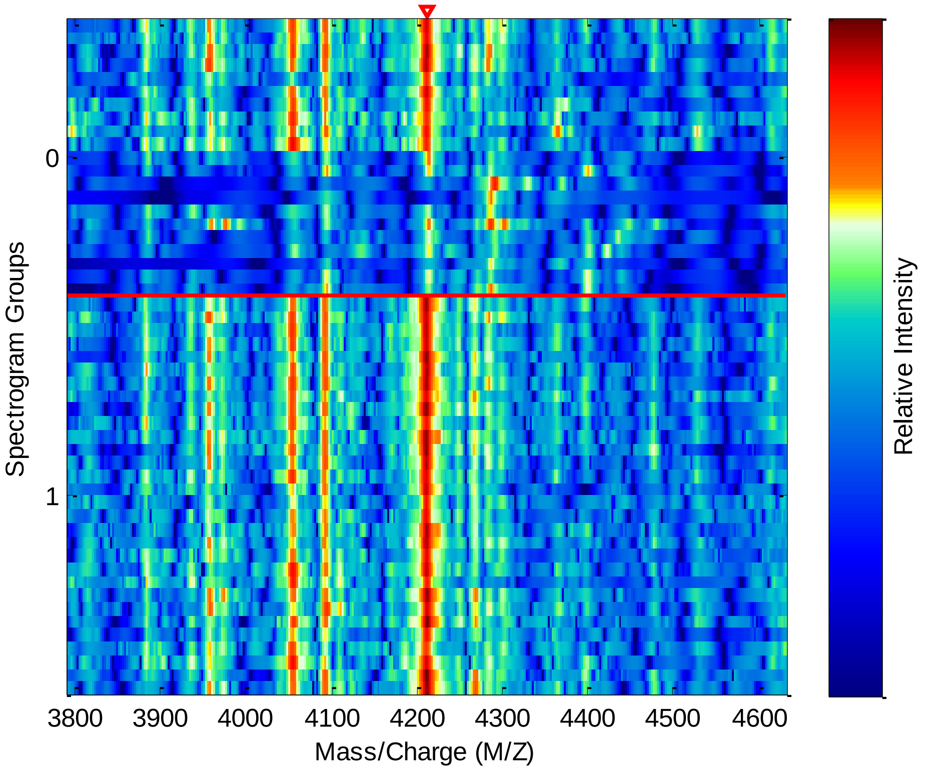

Gel figure: M/Z 4210

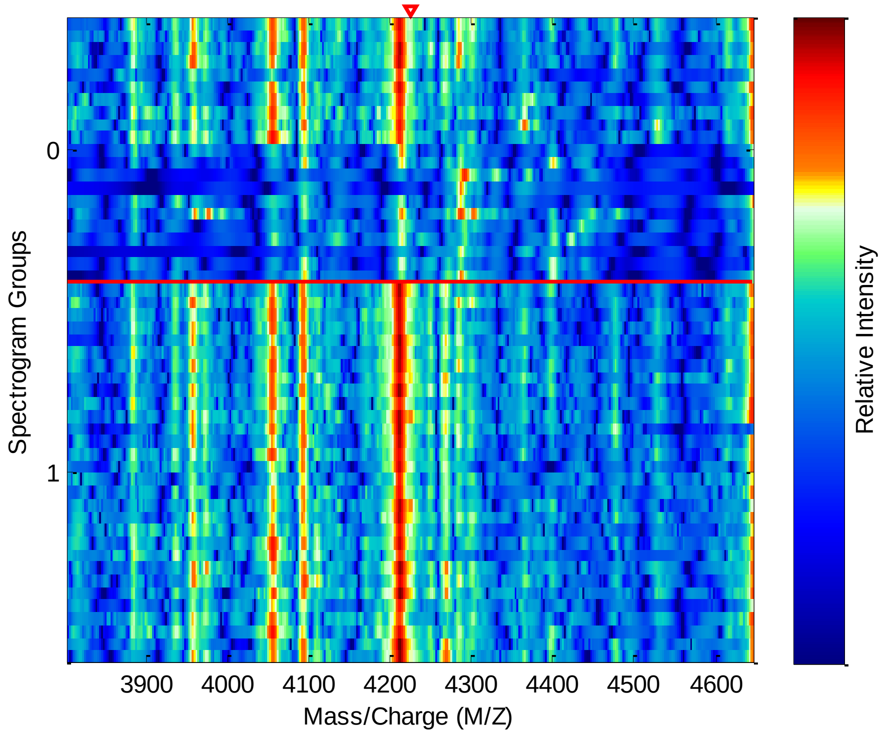

Gel figure: M/Z 4223

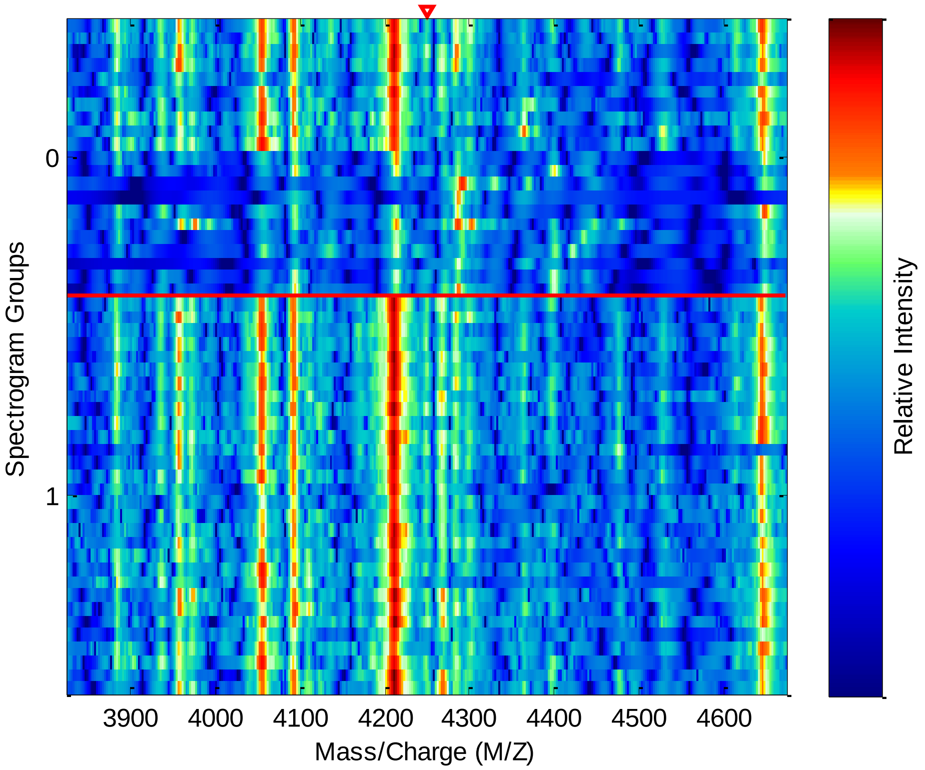

Gel figure: M/Z 4248

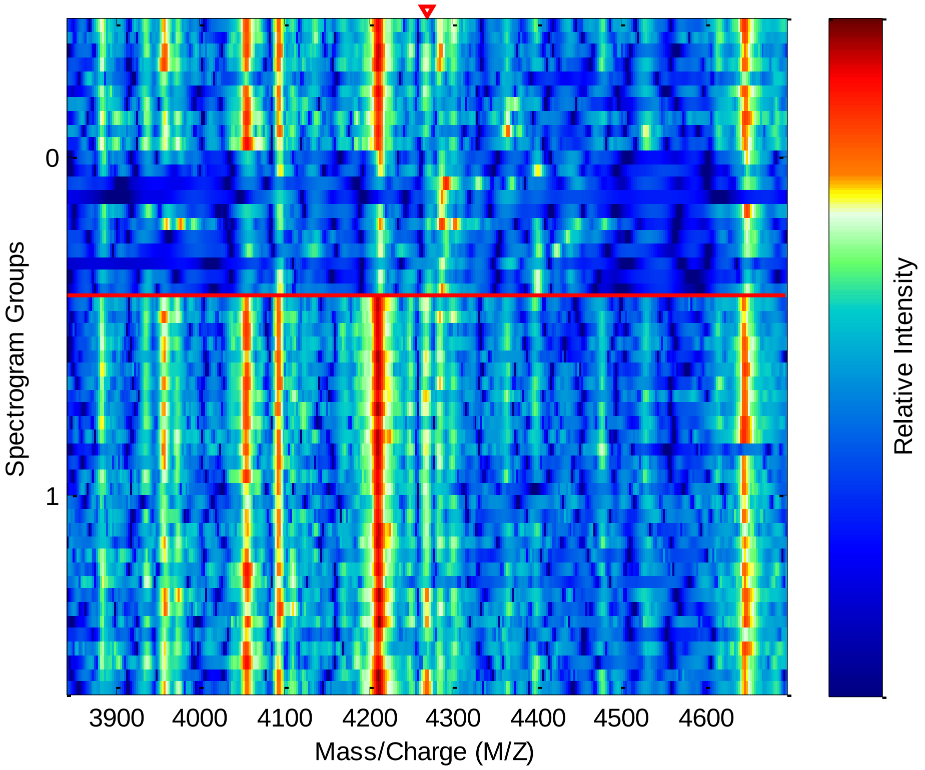

Gel figure: M/Z 4267

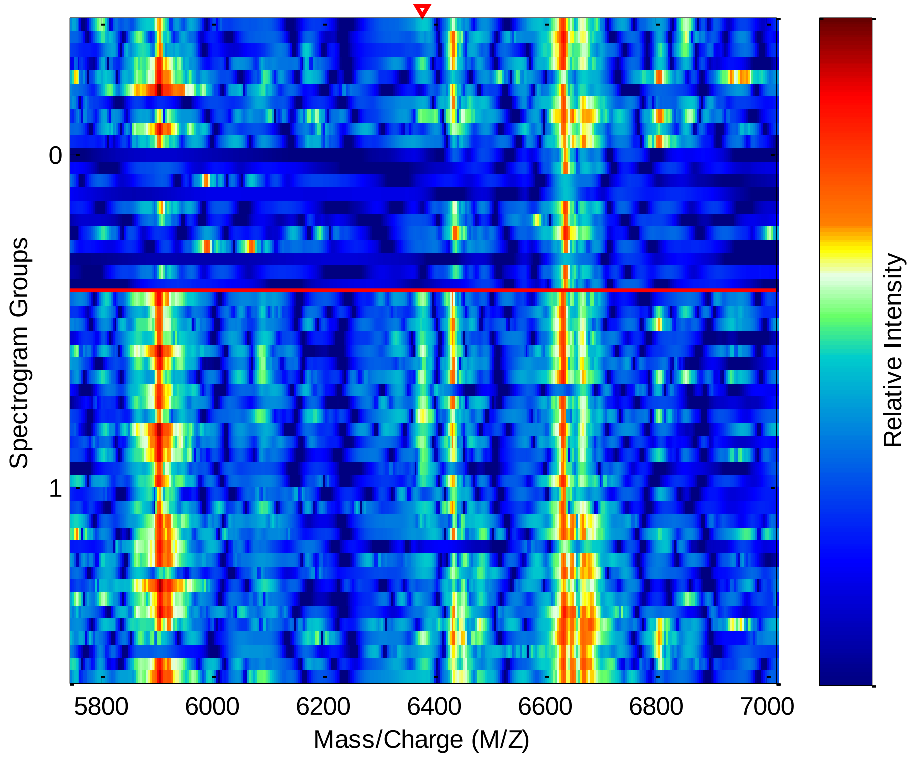

Gel figure: M/Z 6380

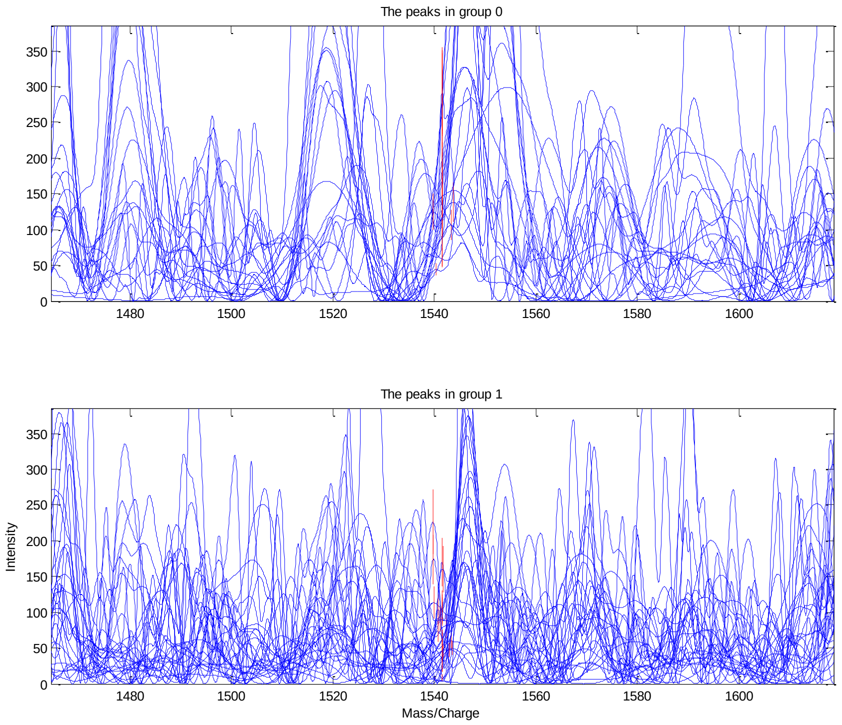

Peak figure: M/Z 1541

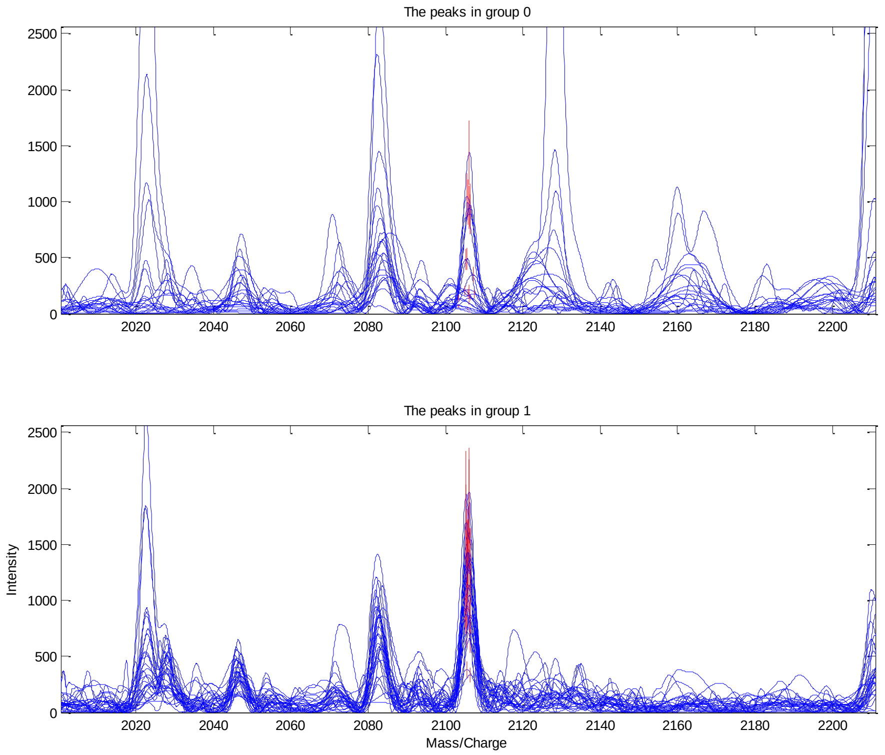

Peak figure: M/Z 2105

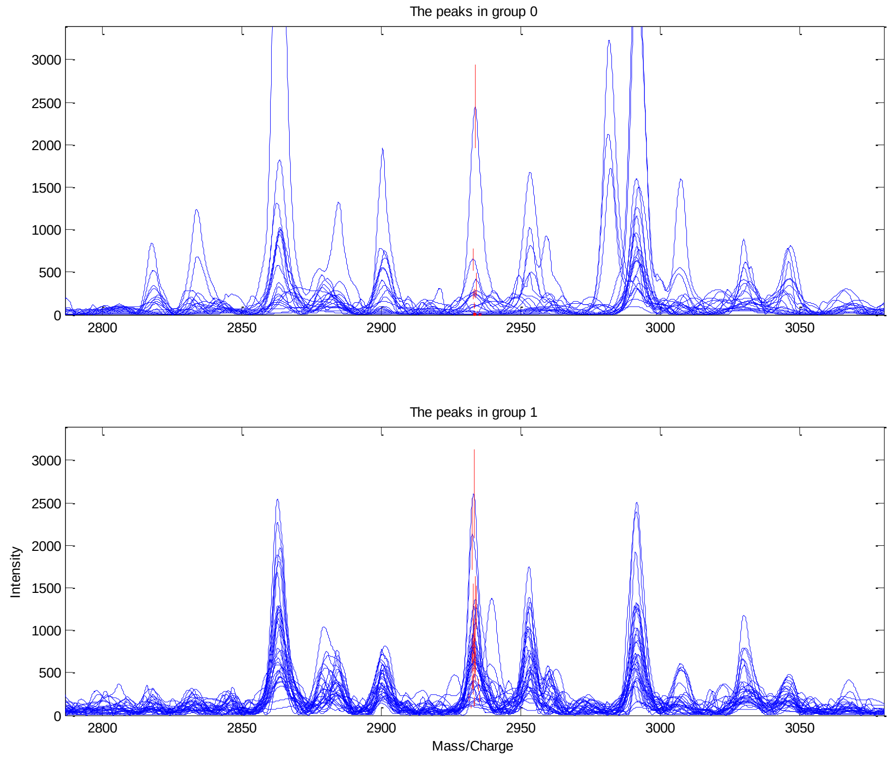

Peak figure: M/Z 2933

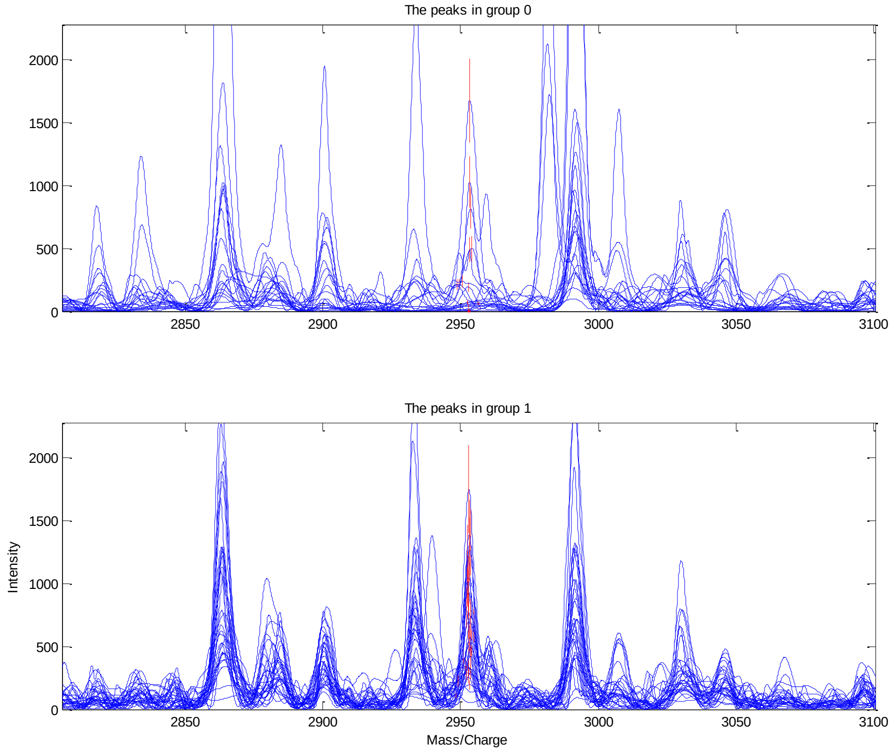

Peak figure: M/Z 2952

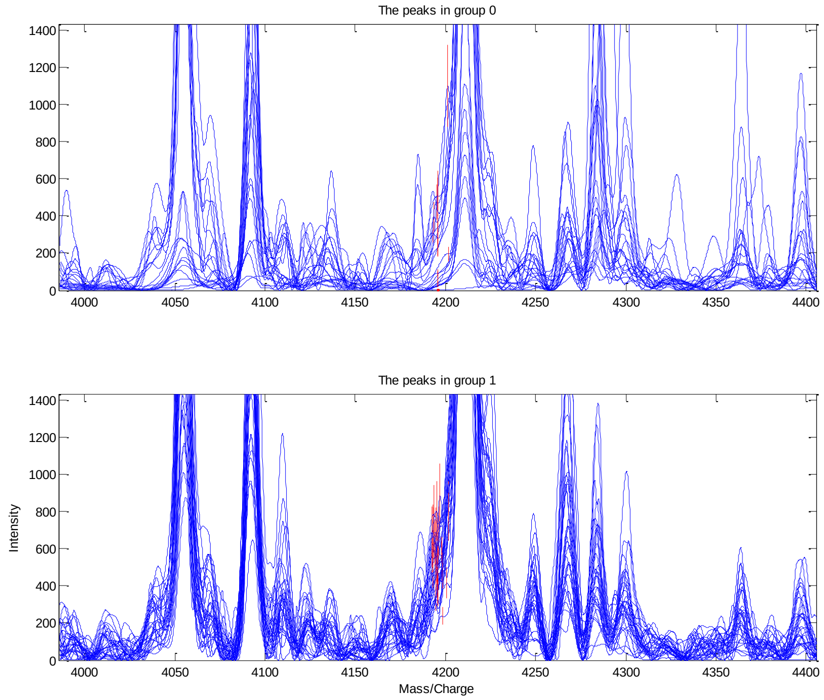

Peak figure: M/Z 4195

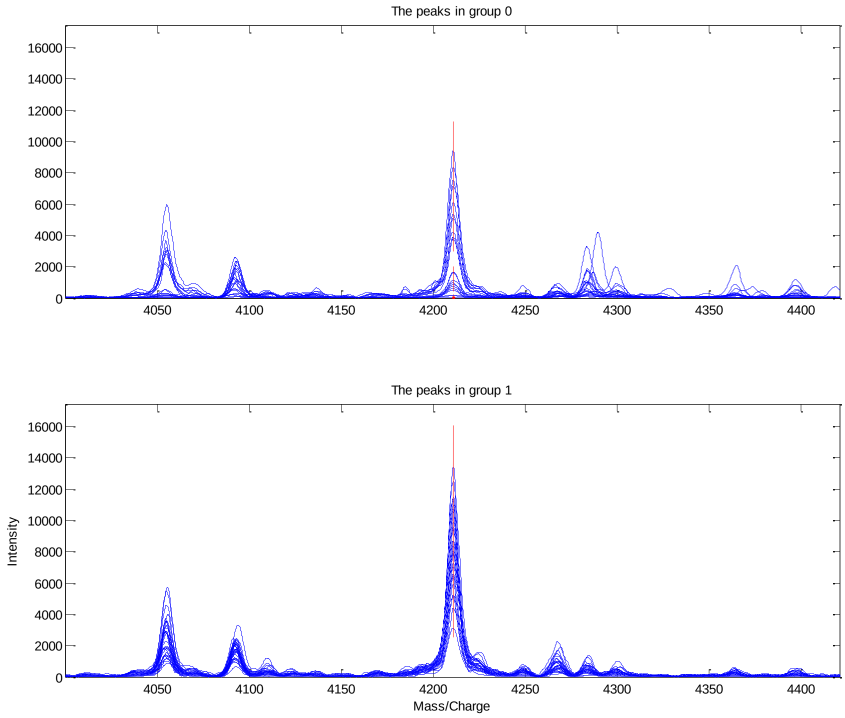

Peak figure: M/Z 4210

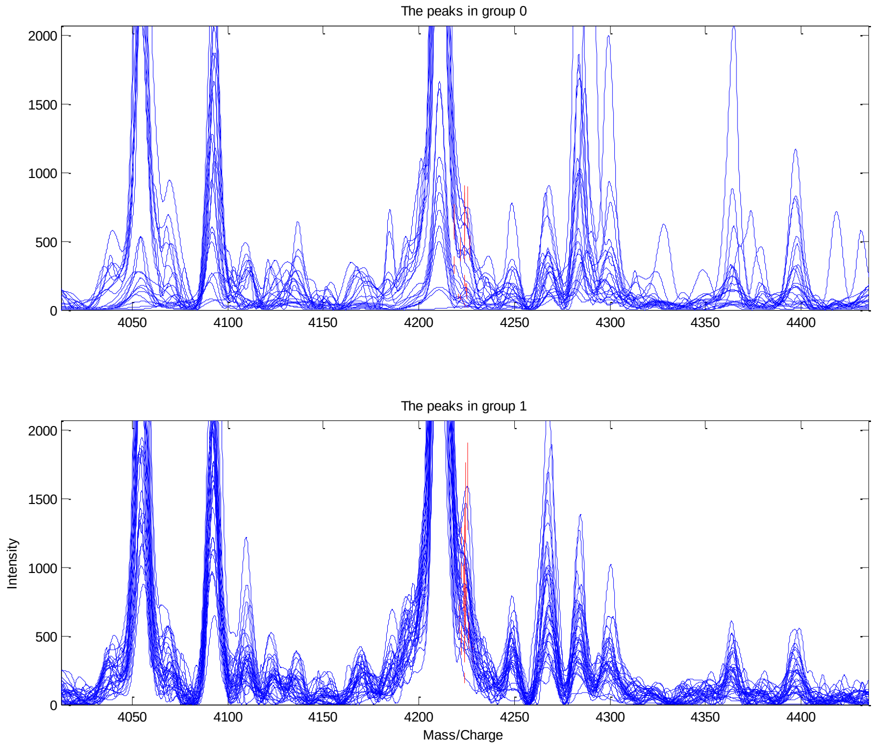

Peak figure: M/Z 4233

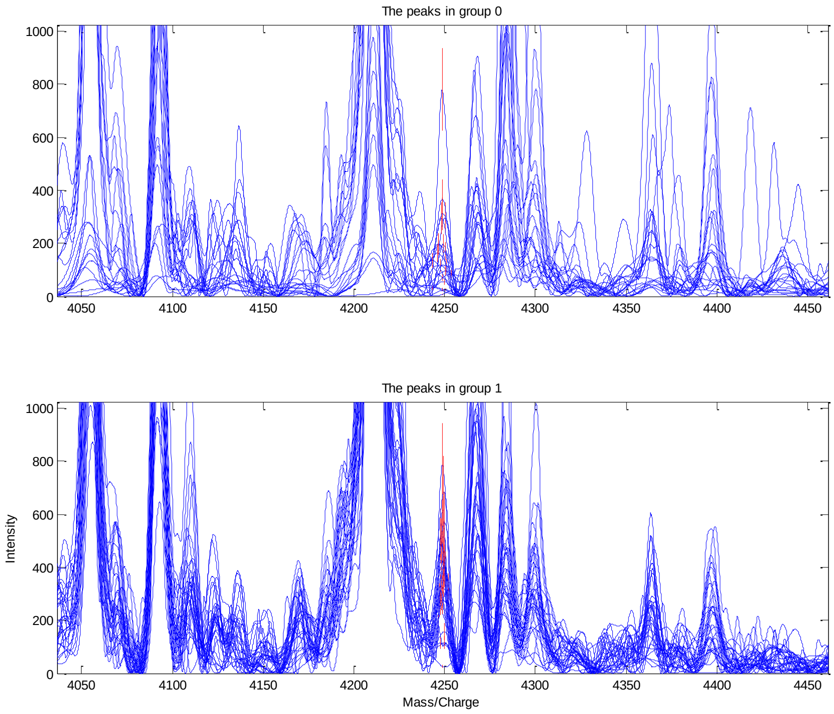

Peak figure: M/Z 4248

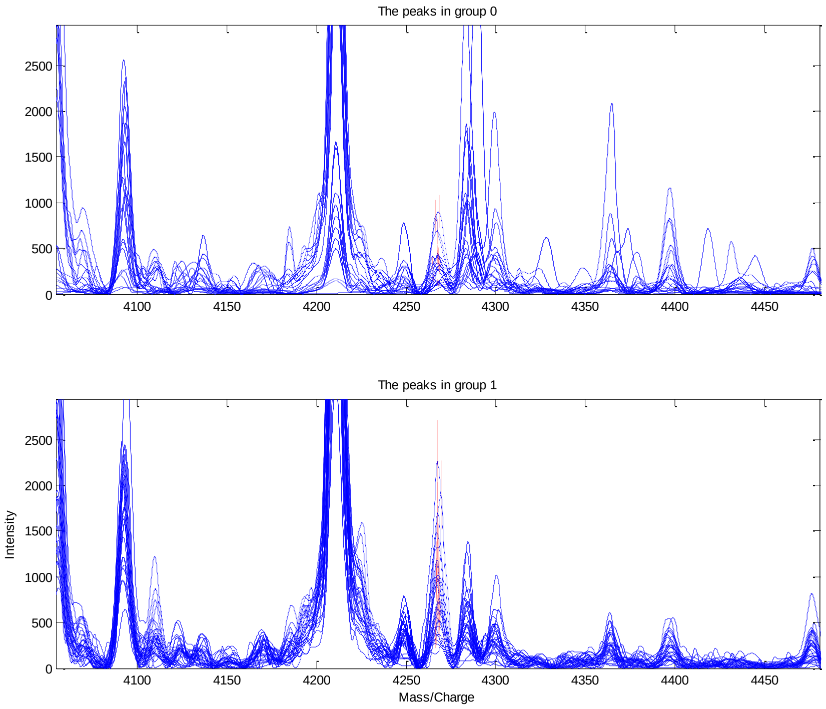

Peak figure: M/Z 4267

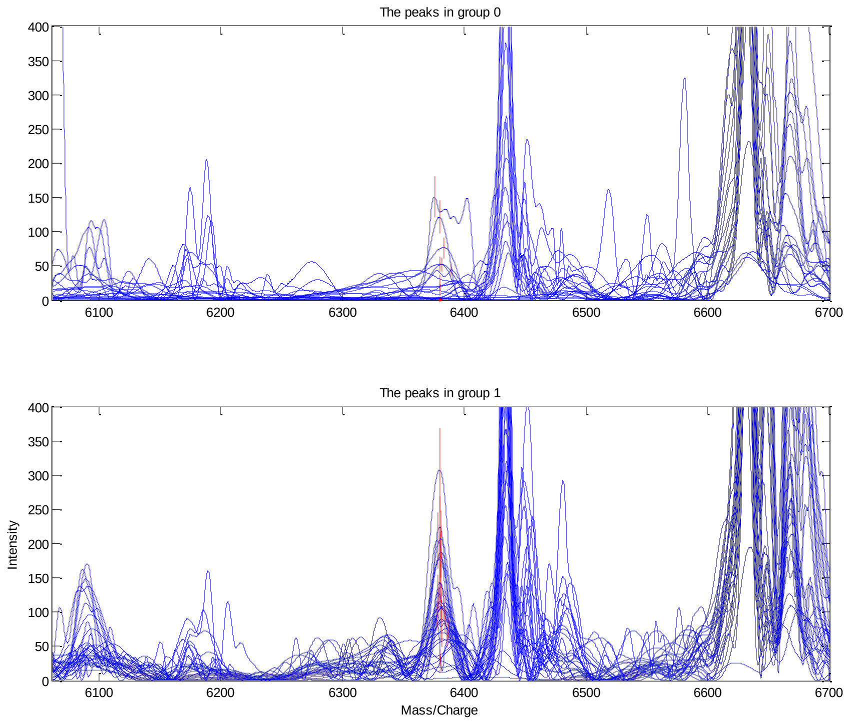

Peak figure: M/Z 6380

User Name	zy2016	Peaks filtering factor	Wilcoxon
Repeat Sample	0	Small Size Sample	No
Test sample percent	0.3	Batch Analyze	1
Wavelet threshold	100	Smoothing window	100
Calibration coefficient (%)	0.03	Minimal M/Z (%)	1000
Cluster factor (%)	0.003	Minimal peak threshold (%)	0.1
Excluded spectra threshold (%)	0.2	Minimal sinal/noise ratio (%)	2
Minimal intensity	300	P value or Number of peaks	10
Algorithm	1	Evaluation	1
GA population	50	GA generation	20

Training result

	Predicted Class 0	Predicted Class 1	Sum	Accuracy (%)	Error (%)
Class 0	18	3	21	85.7143	14.2857
Class 1	2	28	30	93.3333	6.6667

Validation result

	Predicted Class 0	Predicted Class 1	Sum	Accuracy (%)	Error (%)
Class 0	16	5	21	76.1905	23.8095
Class 1	6	24	30	80	20

selected markers is

4267.7998	4223.9247	1541.5624

Biomarker M/Z

4210.7588	4195.7477	4267.7998	2105.8038	2933.4035
4223.9247	6380.9606	2952.8616	4248.7848	1541.5624

P value or Weight

3.4639e-006	5.0034e-006	6.568e-006	7.8604e-006	7.8604e-006
1.2241e-005	1.7336e-005	6.5941e-005	7.147e-005	9.8287e-005

Mean in Class0

3291.1052	239.9033	335.8663	433.0916	237.3828
326.9949	30.1196	276.083	178.0915	153.4355

Mean in Class1

8342.1784	558.4655	853.9418	1185.5369	734.2254
787.9001	108.6418	681.8899	382.4116	.3677

Std in Class0

3046.6524	265.5924	234.2178	428.4893	533.9402
266.3362	41.3966	425.8328	172.3976	77.424

Std in Class1

2548.3934	159.1813	489.673	417.3648	572.2113
329.382	72.4131	416.5696	173.1632	56.1958
